# Interventions for treatment of COVID-19: A living systematic review with meta-analyses and trial sequential analyses (The LIVING Project)

**DOI:** 10.1371/journal.pmed.1003293

**Published:** 2020-09-17

**Authors:** Sophie Juul, Emil Eik Nielsen, Joshua Feinberg, Faiza Siddiqui, Caroline Kamp Jørgensen, Emily Barot, Niklas Nielsen, Peter Bentzer, Areti Angeliki Veroniki, Lehana Thabane, Fanlong Bu, Sarah Klingenberg, Christian Gluud, Janus Christian Jakobsen

**Affiliations:** 1 Copenhagen Trial Unit–Centre for Clinical Intervention Research, Rigshospitalet, Copenhagen University Hospital, Copenhagen, Denmark; 2 Department of Internal Medicine–Cardiology Section, Holbæk Hospital, Holbæk, Denmark; 3 Lund University, Helsingborg Hospital, Department of Clinical Sciences Lund, Anesthesia & Intensive Care, Lund, Sweden; 4 Department of Primary Education, School of Education, University of Ioannina, Ioannina, Greece; 5 Knowledge Translation Program, Li Ka Shing Knowledge Institute, St. Michael’s Hospital, Toronto, Ontario, Canada; 6 Department of Health Research Methods, Evidence, and Impact, McMaster University, Hamilton, Ontario, Canada; 7 Centre for Evidence-based Chinese Medicine, Beijing University of Chinese Medicine, Beijing, China; 8 Faculty of Health Sciences, University of Southern Denmark, Odense, Denmark; University of Palermo, ITALY

## Abstract

**Background:**

Coronavirus disease 2019 (COVID-19) is a rapidly spreading disease that has caused extensive burden to individuals, families, countries, and the world. Effective treatments of COVID-19 are urgently needed.

**Methods and findings:**

This is the first edition of a living systematic review of randomized clinical trials comparing the effects of all treatment interventions for participants in all age groups with COVID-19. We planned to conduct aggregate data meta-analyses, trial sequential analyses, network meta-analysis, and individual patient data meta-analyses. Our systematic review is based on Preferred Reporting Items for Systematic Reviews and Meta-Analysis (PRISMA) and Cochrane guidelines, and our 8-step procedure for better validation of clinical significance of meta-analysis results. We performed both fixed-effect and random-effects meta-analyses. Primary outcomes were all-cause mortality and serious adverse events. Secondary outcomes were admission to intensive care, mechanical ventilation, renal replacement therapy, quality of life, and nonserious adverse events. We used Grading of Recommendations Assessment, Development and Evaluation (GRADE) to assess the certainty of evidence. We searched relevant databases and websites for published and unpublished trials until August 7, 2020. Two reviewers independently extracted data and assessed trial methodology.

We included 33 randomized clinical trials enrolling a total of 13,312 participants. All trials were at overall high risk of bias. We identified one trial randomizing 6,425 participants to dexamethasone versus standard care. This trial showed evidence of a beneficial effect of dexamethasone on all-cause mortality (rate ratio 0.83; 95% confidence interval [CI] 0.75–0.93; *p* < 0.001; low certainty) and on mechanical ventilation (risk ratio [RR] 0.77; 95% CI 0.62–0.95; *p* = 0.021; low certainty). It was possible to perform meta-analysis of 10 comparisons. Meta-analysis showed no evidence of a difference between remdesivir versus placebo on all-cause mortality (RR 0.74; 95% CI 0.40–1.37; *p* = 0.34, I^2^ = 58%; 2 trials; very low certainty) or nonserious adverse events (RR 0.94; 95% CI 0.80–1.11; *p* = 0.48, I^2^ = 29%; 2 trials; low certainty). Meta-analysis showed evidence of a beneficial effect of remdesivir versus placebo on serious adverse events (RR 0.77; 95% CI 0.63–0.94; *p* = 0.009, I^2^ = 0%; 2 trials; very low certainty) mainly driven by respiratory failure in one trial.

Meta-analyses and trial sequential analyses showed that we could exclude the possibility that hydroxychloroquine versus standard care reduced the risk of all-cause mortality (RR 1.07; 95% CI 0.97–1.19; *p* = 0.17; I^2^ = 0%; 7 trials; low certainty) and serious adverse events (RR 1.07; 95% CI 0.96–1.18; *p* = 0.21; I^2^ = 0%; 7 trials; low certainty) by 20% or more, and meta-analysis showed evidence of a harmful effect on nonserious adverse events (RR 2.40; 95% CI 2.01–2.87; *p* < 0.00001; I^2^ = 90%; 6 trials; very low certainty). Meta-analysis showed no evidence of a difference between lopinavir–ritonavir versus standard care on serious adverse events (RR 0.64; 95% CI 0.39–1.04; *p* = 0.07, I^2^ = 0%; 2 trials; very low certainty) or nonserious adverse events (RR 1.14; 95% CI 0.85–1.53; *p* = 0.38, I^2^ = 75%; 2 trials; very low certainty). Meta-analysis showed no evidence of a difference between convalescent plasma versus standard care on all-cause mortality (RR 0.60; 95% CI 0.33–1.10; *p* = 0.10, I^2^ = 0%; 2 trials; very low certainty). Five single trials showed statistically significant results but were underpowered to confirm or reject realistic intervention effects.

None of the remaining trials showed evidence of a difference on our predefined outcomes. Because of the lack of relevant data, it was not possible to perform other meta-analyses, network meta-analysis, or individual patient data meta-analyses. The main limitation of this living review is the paucity of data currently available. Furthermore, the included trials were all at risks of systematic errors and random errors.

**Conclusions:**

Our results show that dexamethasone and remdesivir might be beneficial for COVID-19 patients, but the certainty of the evidence was low to very low, so more trials are needed. We can exclude the possibility of hydroxychloroquine versus standard care reducing the risk of death and serious adverse events by 20% or more. Otherwise, no evidence-based treatment for COVID-19 currently exists. This review will continuously inform best practice in treatment and clinical research of COVID-19.

## Abstract 2

Sophie Juul and colleagues initiate a living systematic review including meta-analyses on treatments for COVID-19.

## Author summary

**Why was this study done?:**

- Severe acute respiratory syndrome coronavirus 2 (SARS-CoV-2) infection has spread rapidly worldwide, causing an international outbreak of the coronavirus disease 2019 (COVID-19).
- There is a need for a living systematic review evaluating the beneficial and harmful effects of all possible interventions for treatment of COVID-19.

**What did the researchers do and find?:**

- We conducted the first edition of a living systematic review with meta-analyses and trial sequential analyses to compare the effects of all treatment interventions for COVID-19.
- One single trial randomizing 6,425 patients showed evidence of a beneficial effect of dexamethasone versus standard care on all-cause mortality and mechanical ventilation.
- Meta-analysis of 2 trials showed evidence of a beneficial effect of remdesivir versus placebo on serious adverse events, but we found no effects on all-cause mortality or adverse events considered not serious.
- Meta-analysis of 6 trials showed evidence of a harmful effect of hydroxychloroquine on adverse events considered nonserious and did not seem to have any effect on all-cause mortality or serious adverse events.
- Five single trials showed statistically significant results but were underpowered to confirm or reject realistic intervention effects.
- None of the remaining trials showed evidence of a difference of the experimental interventions on our predefined outcomes.

**What do these findings mean?:**

- No certain evidence-based treatment is currently available for COVID-19 patients.
- Dexamethasone may reduce all-cause mortality and need for mechanical ventilation in COVID-19 patients.
- Remdesivir may reduce serious adverse events in COVID-19 patients, but the clinical effects and the optimal treatment duration need confirmation.
- More high-quality, low risk of bias randomized clinical trials are urgently needed.

## Introduction

In 2019, a novel coronavirus named severe acute respiratory syndrome coronavirus 2 (SARS-CoV-2) caused an international outbreak of the respiratory illness (coronavirus disease 2019 [COVID-19]) [1]. Since the initial outbreak in China, SARS-CoV-2 has spread globally, and COVID-19 is labeled a public health emergency of global concern by the World Health Organization [2]. The full spectrum of COVID-19 ranges from subclinical infection over mild, self-limiting respiratory tract illness to severe progressive pneumonia, multiorgan failure, and death [3]. Severe disease onset might result in death because of massive alveolar damage and progressive respiratory failure [4–6].

No evidence-based treatment for COVID-19 currently exist to augment widely used supportive care protocols [7]. To control the growing COVID-19 pandemic, we rely on quarantine, isolation, and infection-control measures to prevent disease spread [7] and on supportive care, including oxygen and mechanical ventilation, for infected patients. Many randomized clinical trials assessing the effects of different potential treatments for COVID-19 are currently underway. However, a single trial can rarely validly assess the effects of any intervention, and there is an urgent need to continuously surveil and update the aggregated evidence base so that effective interventions, if such exist, are implemented in clinical practice [8].

The present living systematic review with aggregate meta-analyses and trial sequential analyses aims to continuously inform evidence-based guideline recommendations for the treatment of COVID-19, taking risks of systematic errors (“bias”), risks of random errors (“play of chance”), and certainty of the findings into consideration [9].

## Methods

We report this systematic review based on the Preferred Reporting Items for Systematic Reviews and Meta-Analysis (PRISMA) guidelines (**S1 Text**) [10,11]. The updated methodology used in this living systematic review is described in detail in The Cochrane Handbook of Systematic Reviews of Interventions [12] and our protocol [9], which was registered in the PROSPERO database (ID: CRD42020178787) prior to the systematic literature search.

### Search strategy and selection criteria

#### Electronic searches

An information specialist searched the Cochrane Central Register of Controlled Trials (CENTRAL) in The Cochrane Library, Medical Literature Analysis and Retrieval System Online (MEDLINE Ovid), Excerpta Medica database (Embase Ovid), Latin American and Caribbean Health Sciences Literature (LILACS; Bireme), Science Citation Index Expanded (SCI-EXPANDED; Web of Science), Conference Proceedings Citation Index–Science (CPCI-S; Web of Science), BIOSIS (Web of Science), CINAHL (EBSCO host), Chinese Biomedical Literature Database (CBM), China Network Knowledge Information (CNKI), Chinese Science Journal Database (VIP), and Wafang Database to identify relevant trials. We searched all databases from their inception and until August 7, 2020. Trials were included irrespective of language, publication status, publication year, and publication type. For the detailed search strategies for all electronic searches, see S2 Text.

#### Searching other resources

The reference lists of relevant trial publications were checked for any unidentified randomized clinical trials. To identify unpublished trials, we searched clinical trial registries (e.g., clinicaltrials.gov, clinicaltrialregister.eu, who.int/ictrp, chictr.org.cn) of Europe, USA, and China, and websites of pharmaceutical companies, websites of US Food and Drug Administration (FDA) and European Medicines Agency (EMA). We also searched the COVID-19 Study Registry [13] and the real-time dashboard of randomized trials [14].

We included unpublished and grey literature trials and assessed relevant retraction statements and errata for included trials. We also searched preprint servers (bioRxiv, medRxiv) for unpublished trials. We contacted all trial authors to obtain individual patient data.

### Living systematic review

In this living systematic review, 2 independent investigators receive a weekly updated literature search file and continuously include relevant newly published or unpublished trials. The relevant meta-analyses, trial sequential analyses, and network meta-analysis will be continuously updated, and if new evidence is available (judged by the author group), the results will be submitted for publication. Every month, the author group will discuss whether searching once a week is necessary. For a detailed overview of the living systematic review work flow, see our protocol [9]. As this is a living systematic review analyzing results of randomized clinical trials, no ethical approval is required.

### Data extraction

Two authors (EEN and JF) independently screened relevant trials. Six authors in pairs (SJ, EEN, JF, FS, CKJ, EB) independently extracted data using a standardized data extraction sheet. Any discrepancies were resolved through discussion, or if required, through discussion with a third author (JCJ). We contacted all trial authors if relevant data were unclear or missing.

### Risk of bias assessment

Risk of bias was assessed with the Cochrane Risk of Bias tool–version 2 (RoB 2) [12,15]. Six authors in pairs (SJ, EEN, JF, FS, CKJ, EB) independently assessed risk of bias. Any discrepancies were resolved through discussion or, if required, through discussion with a third author (JCJ). Bias was assessed with the following domains: bias arising from the randomization process, bias due to deviations from the intended interventions, bias due to missing outcome data, bias in measurement of outcomes, and bias arising from selective reporting of results [12,15]. We contacted authors of trials with unclear or missing data.

### Outcomes and subgroup analyses

Primary and secondary outcomes were predefined in our protocol [9]. Primary outcomes were all-cause mortality and serious adverse events (as defined by the International Conference on Harmonation–Good Clinical Practice [ICH-GCP] guidelines) [9,16]. Secondary outcomes were admission to intensive care (as defined by trialists), receipt of mechanical ventilation (as defined by trialists), receipt of renal replacement therapy (as defined by trialists), quality of life, and nonserious adverse events. We classified nonserious adverse events as any adverse event not assessed as serious according to the ICH-GCP definition.

We chose to add time to clinical improvement as a post hoc outcome. We planned several subgroup analyses, which are described in detail in our protocol [9]. For all outcomes, we used the trial results reported at maximum follow-up.

### Assessment of statistical and clinical significance

We performed our aggregate data meta-analyses according to Cochrane [12], Keus and colleagues [17], and the 8-step assessment by Jakobsen and colleagues [18] for better validation of meta-analytic results in systematic reviews. Review Manager version 5.4 (The Nordic Cochrane Centre, The Cochrane Collaboration, Copenhagen, Denmark) [19] and Stata 16 (StataCorp LLC, College Station, TX, USA) [20] were used for all statistical analyses. We used risk ratios (RRs) for dichotomous outcomes. We planned to calculate the mean differences (MDs) and standardized mean difference (SMD) with 95% confidence intervals (CI) for continuous outcomes. We assessed a total of 2 primary outcomes, and we therefore adjusted our thresholds for significance [18] and considered a *p*-value of 0.033 or less as the threshold for statistical significance [9,18]. Because we primarily considered results of secondary outcomes as hypothesis generating, we did not adjust the *p*-value for secondary outcomes. We conducted both random-effects (Inverse Variance, DerSimonian-Laird) and fixed-effect (Mantel–Haenszel) meta-analyses for all analyses and chose the most conservative result as our primary result [12,18,21,22]. We used trial sequential analysis to control for random errors [23–31]. Trial sequential analysis estimates the diversity-adjusted required information size (DARIS), which is the number of participants needed in a meta-analysis to detect or reject a certain intervention effect. Statistical heterogeneity was quantified by calculating inconsistency (I square) for traditional meta-analyses and diversity (D square) for trial sequential analysis. We used GRADE to assess the certainty of evidence. We downgraded imprecision in GRADE by 2 levels if the accrued number of participants were below 50% of the DARIS and one level if between 50% and 100% of DARIS. We did not downgrade if benefit, harm, futility, or DARIS were reached. Trial sequential analysis will also be used in future updates to adjust the thresholds for significance according to repetitive testing. We used Fisher’s exact test to calculate *p*-values for all single trial results.

## Results

### Study characteristics

**Fig 1:**
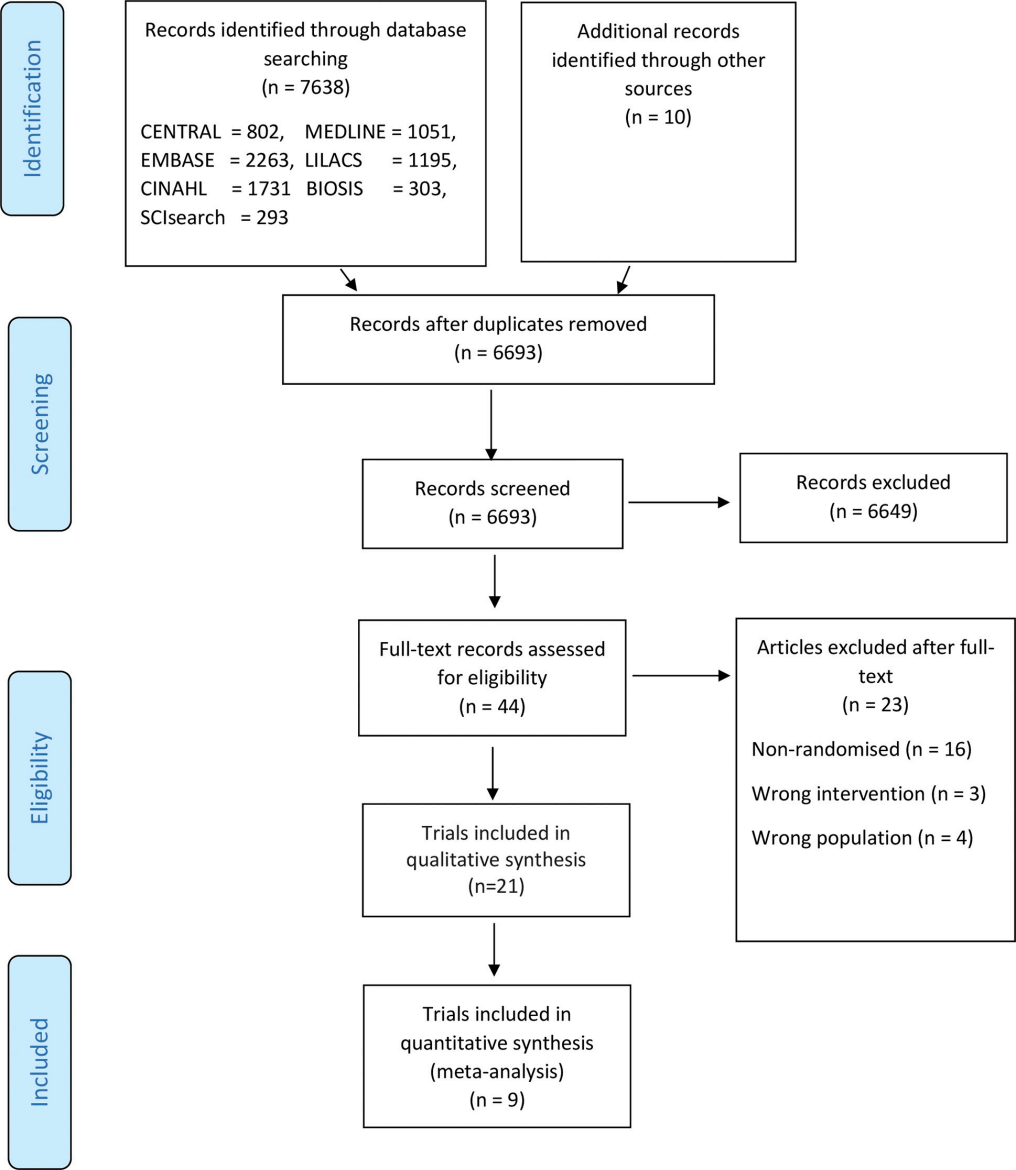
PRISMA flow diagram. BIOSIS, Biosciences Information Services; CENTRAL, Cochrane Central Register of Controlled Trials; CINAHL, Current Index to Nursing and Allied Health Literature; EMBASE, Excerpta Medica database; LILCAS, Latin American and Caribbean Health Sciences Literature; MEDLINE, Medical Literature Analysis and Retrieval System Online; PRISMA, Preferred Reporting Items for Systematic Reviews and Meta-Analysis; SCIsearch, Science Citation Index Search.

On August 7, 2020, our literature searches identified 8,082 records after duplicates were removed. We included a total of 33 clinical trials randomizing 13,312 participants **(Fig 1**) [32–65]. We identified several trials, including participants suspected of COVID-19 [66,67]. None of the trials reported separate data on COVID-19 positive participants compared to the remaining participants. We included trials if approximately 50% or more participants had a confirmed COVID-19 diagnosis. We wrote to all authors requesting separate data on COVID-19 confirmed participants, but we have received no responses yet. For at detailed overview of excluded trials, see **S1 Table.**

Characteristics of included trials and the trial results can be found in **S2 Table**. All trials were assessed as at high risk of bias (**S3 Table**). One trial compared dexamethasone versus standard care [48,53]. Two trials compared remdesivir versus placebo [32,44]. Two trials compared lopinavir–ritonavir added to standard care versus standard care alone [33,41]. Eight trials compared hydroxychloroquine added to standard care versus standard care alone [35,36,43,49,55,56,59,60]. Two trials compared convalescent plasma added to standard care versus standard care alone [40,52]. The remaining trials and comparisons included hydroxychloroquine with and without azithromycin added to standard care versus standard care alone [55], hydroxychloroquine versus placebo [54,68], hydroxychloroquine and chloroquine added to standard care versus standard care alone [49], methylprednisolone added to standard care versus standard care alone [57], lopinavir–ritonavir versus umifenovir and versus standard care [41], favipravir versus umifenovir [34], high-flow nasal oxygenation versus standard mask oxygenation prior to fibreotic tracheal intubation [45], α-lipoic acid versus placebo [47], the combination of novaferon plus lopinavir–ritonavir versus novaferon and versus lopinavir–ritonavir [46], baloxavir marboxil versus favipiravir and versus standard care [42], 5 versus 10 days of remdesivir [38], interferon β-1a added to standard care versus standard care alone [37], colchicine added to standard care versus standard care alone [50], high-dosage with low-dosage chloroquine diphosphate [51], intravenous immunoglobulin added to standard care versus standard care alone [58], ribavirin plus interferon alpha versus lopinavir/ritonavir plus interferon alpha versis ribavirin plus lopinavir/ritonavir plus interferon alpha [62], darunavir/cobicistat plus interferon alpha-2b versus interferon alpha-2b alone [61], lincomycin HCl versus azitromycin [63], 99mTc-methyl diphosphonate (99mTc-MDP) injection added to standard care versus standard care alone [64], and interferon alpha-2b plus gamma versus interferon alpha-2b alone [65].

The maximum follow-up time ranged from 5 [35,36] to 30 [39,58] days after randomization. For several of our outcomes, it was not possible to conduct meta-analysis because of insufficient data.

### Glucocorticosteroids versus standard care

We identified one trial, the Randomised Evaluation of COVid-19 thERapY (RECOVERY) trial, randomizing 6,425 participants to dexamethasone versus standard care [48,53]. Maximum follow-up was 28 days after randomization. The trial was assessed at high risk of bias (**S3 Table**), and the certainty of evidence was assessed at “low” for all-cause mortality, serious adverse events, and mechanical ventilation (**S4 Table**).

- **All-cause mortality:** 482/2,104 died in the dexamethasone group compared with 1,110/4,321 in the standard care group (age-adjusted rate ratio, 0.83; 95% CI 0.75–0.93; *p* < 0.001).
- **Serious adverse events**: 482/2,104 experienced one or more serious adverse events in the dexamethasone group compared with 1,110/4,321 in the standard care group (age-adjusted rate ratio, 0.83; 95% CI 0.75–0.93; *p* < 0.001). This data is based on mortality data only, as suggested by the ICH-GCP definition of a serious adverse event [16].
- **Intensive care**: No data.
- **Mechanical ventilation:** 102/1,780 received invasive mechanical ventilation in the dexamethasone group compared with 285/3,638 in the standard care group (RR 0.77; 95% CI 0.62–0.95).
- **Renal replacement therapy:** No data.
- **Quality of life**: No data.
- **Nonserious adverse events:** No data.

We identified another trial randomizing 63 participants to a different glucocorticoid (methylprednisolone) than the RECOVERY trial [57]. It was not possible to perform meta-analysis, as approximately half of the participants in the experimental group were nonrandomized [57]. We have contacted the trial authors and asked for separate data for all randomized participants, but we have not received a response yet.

### Remdesivir versus placebo

We identified 2 trials comparing remdesivir versus placebo [32,44]. Both trials were assessed as at high risk of bias (**S3 Table**).

### Meta-analysis and trial sequential analysis of all-cause mortality

**Fig 2:**
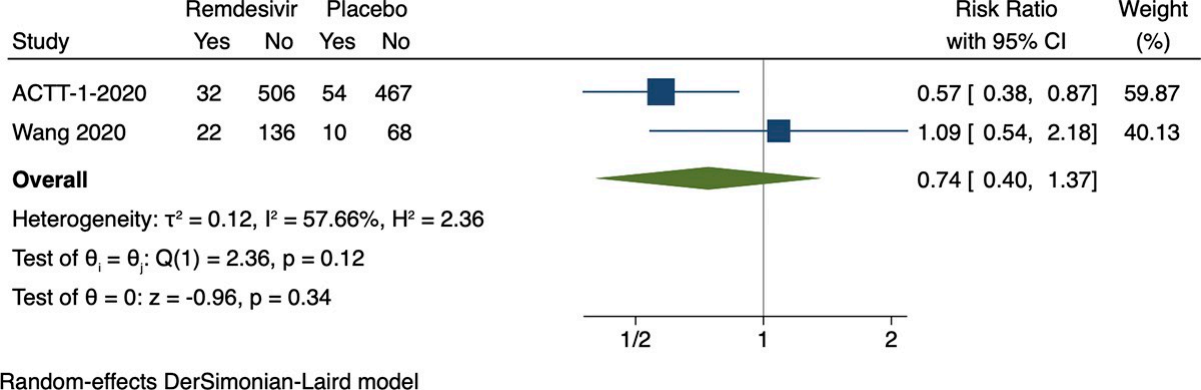
Meta-analysis of remdesivir versus placebo on all-cause mortality. ACTT-1-2020, Adaptive COVID-19 Treatment Trial 1; CI, confidence interval.

**Fig 3:**
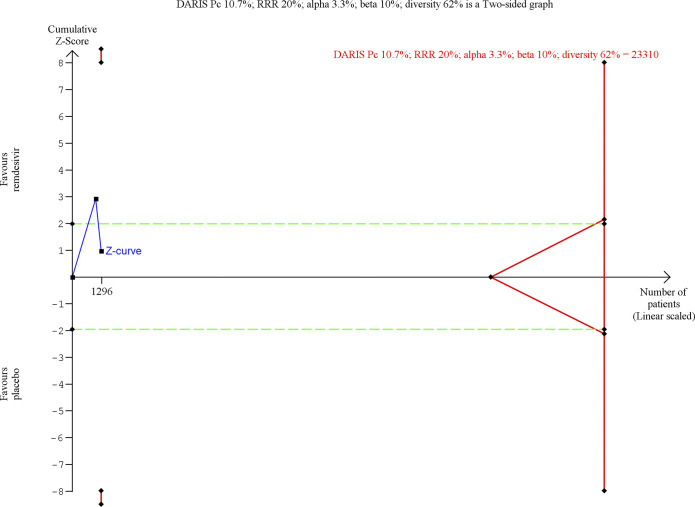
Trial sequential analysis of remdesivir versus placebo on all-cause mortality. Trial sequential analysis on remdesivir versus placebo on all-cause mortality in 2 trials at high risk of bias. The DARIS was calculated based on a mortality proportion in the control group of 10.7%; risk ratio reduction of 20% in the experimental group; type I error of 3.3%; and type II error of 10% (90% power). Diversity was 62%. The required information size was 23,310 participants. The cumulative Z‐curve (blue line) did not cross the trial sequential monitoring boundaries for benefit or harm. The cumulative Z‐curve did not cross the inner‐wedge futility line (red outward sloping lines nor the DARIS). The green dotted line shows conventional boundaries (alpha 5%). DARIS, diversity‐adjusted required information size; Pc, proportion of participants in control group; RRR, relative risk reduction.

Random-effects meta-analysis showed no evidence of a difference between remdesivir versus placebo on all-cause mortality (RR 0.74; 95% CI 0.40–1.37; *p* = 0.34, I^2^ = 58%; 2 trials; very low certainty) (**Fig 2; S5 Table**). Visual inspection of the forest plot and measures to quantify heterogeneity (I^2^ = 58%) indicated heterogeneity. The outcome was assessed 14 days after randomization in the first trial [32] and 28 days after randomization in the second trial [44]. Trial sequential analysis showed that we did not have enough information to confirm or reject that remdesivir versus placebo reduces all-cause mortality with a relative risk reduction of 20% [9] (**Fig 3**). The fixed-effect meta-analysis showed evidence of a beneficial effect of remdesivir versus placebo on all-cause mortality (RR 0.67; 95% CI 0.47–0.96; *p* = 0.03; I^2^ = 58%; 2 trials; very low certainty) (**S1 Fig).**

### Meta-analysis and trial sequential analysis of serious adverse events

**Fig 4:**
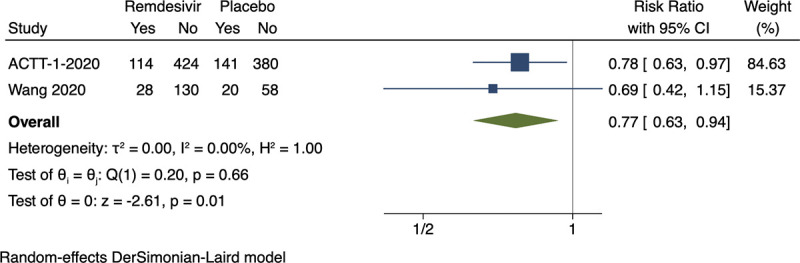
Meta-analysis of remdesivir versus placebo on serious adverse events. ACTT-1-2020, Adaptive COVID-19 Treatment Trial 1; CI, confidence interval.

**Fig 5:**
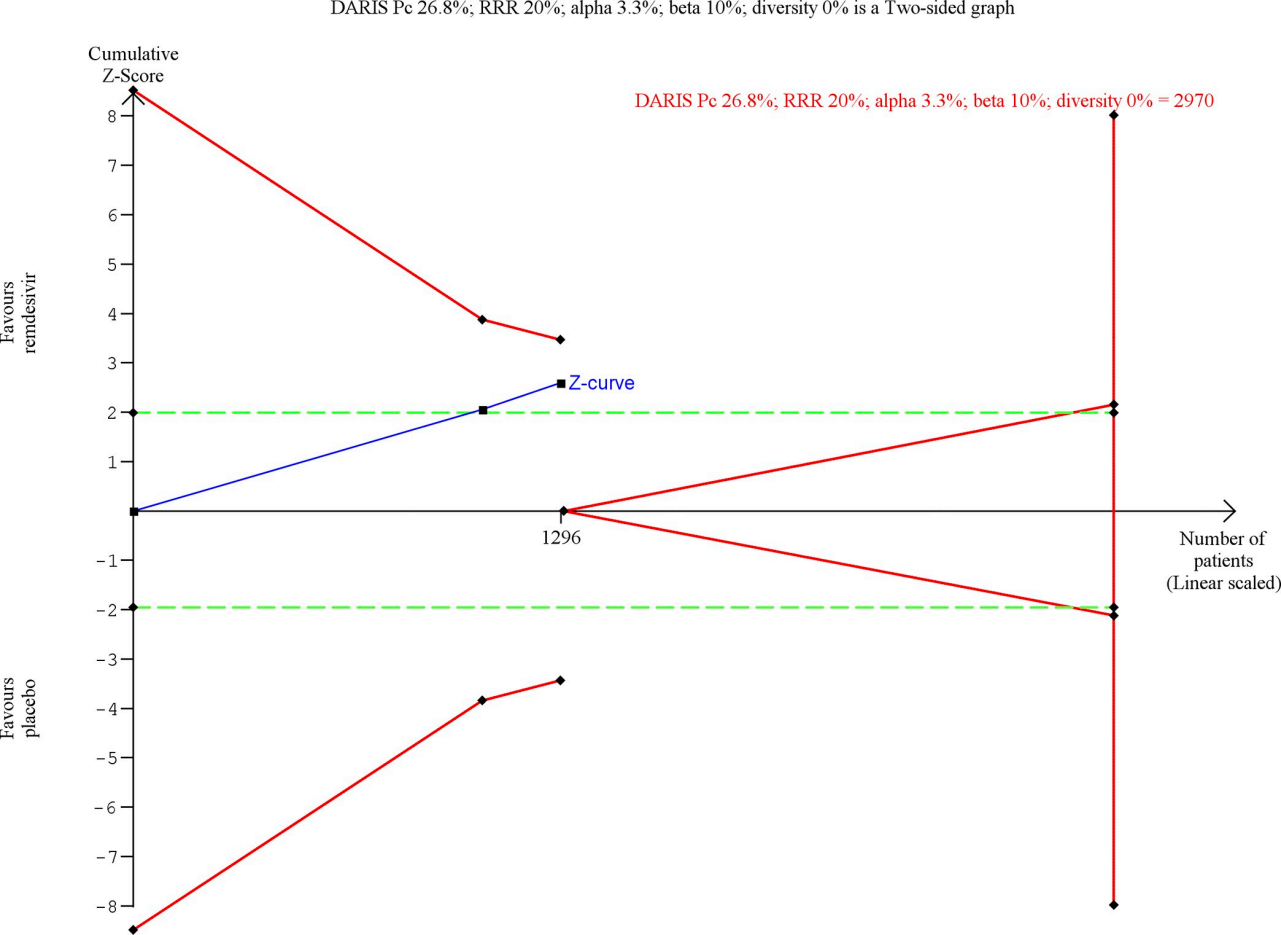
Trial sequential analysis of remdesivir versus placebo on serious adverse events. Trial sequential analysis on remdesivir versus placebo on serious adverse events in 2 trials at high risk of bias. The DARIS was calculated based on a proportion in the control group of 26.8%; risk ratio reduction of 20% in the experimental group; type I error of 3.3%; and type II error of 10% (90% power). Diversity was 0%. The required information size was 2,970 participants. The cumulative Z‐curve (blue line) did not cross the trial sequential monitoring boundaries for benefit or harm (red inward sloping lines). The cumulative Z‐curve did not cross the inner‐wedge futility line (red outward sloping lines nor the DARIS). The green dotted line shows conventional boundaries (alpha 5%). DARIS, diversity‐adjusted required information size; Pc, proportion of participants in control group; RRR, relative risk reduction.

Random-effects meta-analysis showed evidence of a beneficial effect of remdesivir versus placebo on serious adverse events (RR 0.77; 95% CI 0.63–0.94; *p* = 0.009, I^2^ = 0%; 2 trials; very low certainty) (**Fig 4; S5 Table**). Visual inspection of the forest plot and measures to quantify heterogeneity (I^2^ = 0%) indicated no heterogeneity. The outcome was assessed 14 days after randomization in the first trial [32] and 28 days after randomization in the second trial [44]. Trial sequential analysis showed that we did not have enough information to confirm or reject that remdesivir versus placebo reduced the risk of serious adverse events with a relative risk reduction of 20% [9] (**Fig 5**). The difference between groups was mainly driven by a difference in respiratory failure.

### Meta-analysis and trial sequential analysis of nonserious adverse events

Random-effects meta-analysis showed no evidence of a difference between remdesivir versus placebo on adverse events not considered serious (RR 0.94; 95% CI 0.80–1.11; *p* = 0.48, I^2^ = 29%: 2 trials; low certainty) (**S2 Fig; S5 Table**). Visual inspection of the forest plot and measures to quantify heterogeneity (I^2^ = 29%) indicated no major heterogeneity. The outcome was assessed 14 days after randomization in the first trial [32] and 28 days after randomization in the second trial [44]. Trial sequential analysis showed that we had enough information to reject that remdesivir versus placebo reduced the risk of nonserious adverse events with a relative risk reduction of 20% [9] (**S3 Fig**).

### Meta-analysis of time to clinical improvement/recovery

Random-effects meta-analysis showed evidence of a beneficial effects of remdesivir versus placebo on time to clinical improvement/recovery (log ratio of means −0.28; 95% CI −0.55 to −0.02; *p* = 0.04; I^2^ = 0%; 2 trials; very low certainty) (**S4 Fig**). The 2 trials defined this outcome differently. The first trial analyzed “time to recovery” defined as either discharge from the hospital or hospitalization for infection-control purposes only [32]. The second trial analyzed “time to clinical improvement” defined as the time (in days) from randomization to the point of a decline of 2 levels on a 6-point ordinal scale of clinical status (from 1 = discharged to 6 = death) or discharged alive from hospital, whichever came first [44]. Visual inspection of the forest plot and measures to quantify heterogeneity (I^2^ = 0%) indicated no heterogeneity. The outcome was assessed 14 days after randomization in the first trial [32] and 28 days after randomization in the second trial [44].

One trial assessing the effects of remdesivir reported assessment of viral load. The trial result indicated no evidence of a difference from day 1 to day 28 [44].

### Hydroxychloroquine versus standard care

We identified 8 trials comparing hydroxychloroquine added to standard care versus standard care alone [35,36,43,49,55,56,59,60]. We also identified one trial that used placebo as an additional control intervention [54]. All trials were assessed as at high risk of bias (**S3 Table**). One trial was not eligible for meta-analysis, as the results were not reported in a usable way; i.e., the results were reported as per-protocol, and several participants crossed over [43].

### Meta-analysis of all-cause mortality

Fixed-effect meta-analysis showed no evidence of a difference between hydroxychloroquine versus standard care on all-cause mortality (RR 1.07; 95% CI 0.97–1.19; *p* = 0.17; I^2^ = 0%; 7 trials; low certainty) (**S5 Fig, S6 Table).** Visual inspection of the forest plot and measures to quantify heterogeneity (I^2^ = 0%) indicated no heterogeneity. The assessment time points varied from 5 [35] to 28 [49,56,59] days after randomization. The trial sequential analysis showed that we had enough information to reject that hydroxychloroquine compared with standard care reduces all-cause mortality with a relative risk reduction of 20% (**S6 Fig**).

### Meta-analysis of serious adverse events

Fixed-effect meta-analysis showed no evidence of a difference between hydroxychloroquine versus standard care on serious adverse events (RR 1.07; 95% CI 0.96–1.18; *p* = 0.21; I^2^ = 0%; 7 trials; low certainty) (**S7 Fig, S6 Table**). Visual inspection of the forest plot and measures to quantify heterogeneity (I^2^ = 0%) indicated no heterogeneity. The assessment time points varied from 5 [35] to 28 [49,56,59] days after randomization. The trial sequential analysis showed that we had enough information to reject that hydroxychloroquine compared with standard care reduces all-cause mortality with a relative risk reduction of 20% (**S8 Fig**).

### Meta-analysis of nonserious adverse events

Fixed-effect meta-analysis showed evidence of a beneficial effect of standard care on adverse events not considered serious (RR 2.40; 95% CI 2.01–2.87; *p* < 0.00001; I^2^ = 90%; 6 trials; very low certainty) (**S9 Fig, S6 Table**). Visual inspection of the forest plot and measures to quantify heterogeneity (I^2^ = 90%) indicated large heterogeneity. The assessment time points varied from 5 [35,36,54] to 28 [49,56] days after randomization. It was not possible to perform trial sequential analysis due to high diversity [9].

### Lopinavir–ritonavir versus standard care

We identified 2 trials comparing lopinavir–ritonavir added to standard care versus standard care alone [33,41]. Both trials were assessed as at high risk of bias (**S3 Table**).

### Meta-analysis and trial sequential analysis of serious adverse events

Random-effects meta-analysis showed no evidence of a difference between lopinavir–ritonavir versus standard care on serious adverse events (RR 0.64; 95% CI 0.39–1.04; *p* = 0.07, I^2^ = 0%; 2 trials; very low certainty) (**S10 Fig, S7 Table)**. Visual inspection of the forest plot and measures to quantify heterogeneity (I^2^ = 0%) indicated no heterogeneity. The assessment time point was 21 days after randomization in the first trial [41] and 28 days after randomization in the second trial [33]. Trial sequential analysis showed that we did not have enough information to confirm or reject that lopinavir–ritonavir versus standard care reduced the risk of serious adverse events with a relative risk reduction of 20% [9] (**S11 Fig**).

### Meta-analysis of nonserious adverse events

Random-effects meta-analysis showed no evidence of a difference between lopinavir–ritonavir versus standard care on adverse events not considered as serious (RR 1.14; 95% CI 0.85–1.53; *p* = 0.38, I^2^ = 75%; 2 trials; very low certainty) (**S12 Fig; S7 Table**). Visual inspection of the forest plot and measures to quantify heterogeneity (I^2^ = 75%) indicated substantial heterogeneity. The assessment time point was 21 days after randomization in the first trial [41] and 28 days after randomization in the second trial [33]. Trial sequential analysis showed that we did not have enough information to confirm or reject that lopinavir–ritonavir compared with standard care reduces nonserious adverse events with a relative risk reduction of 20% [9].

### Convalescent plasma versus standard care

We identified 2 trials comparing convalescent plasma added to standard care versus standard care alone [40,52]. Both trials were assessed as at high risk of bias (**S3 Table**).

### Meta-analysis of all-cause mortality

Random-effects meta-analysis showed no evidence of a difference between convalescent plasma versus standard care on all-cause mortality (RR 0.60; 95% CI 0.33–1.10; *p* = 0.10, I^2^ = 0%; 2 trials; very low certainty) (**S13 Fig; S8 Table**). Visual inspection of the forest plot and measures to quantify heterogeneity (I^2^ = 0%) indicated no heterogeneity. The outcome was assessed 15 days after randomization in the first trial [52] and 28 days after randomization in the second trial [40]. Trial sequential analysis showed that we did not have enough information to confirm or reject that convalescent plasma reduces all-cause mortality with a relative risk reduction of 20% [9].

### Remaining trial data

Because of a lack of relevant data, it was not possible to conduct other meta-analyses, individual patient data meta-analyses, or network meta-analysis. Five single trials showed statistically significant results but were underpowered to confirm or reject realistic intervention effects. One trial randomizing 402 participants compared 5 versus 10 days of remdesivir showed evidence of a beneficial effect of 5 days of remdesivir on serious adverse events (*p* = 0.003 [Fisher’s exact test]) [38]. One trial randomizing 92 participants compared the immunomodulator interferon β-1a added to standard care versus standard care alone showed evidence of a beneficial effect of interferon β-1a on all-cause mortality (*p* = 0.029) [37]. This trial also showed evidence of a beneficial effect of standard care on nonserious adverse events (*p* = 0.006) [37]. One single trial randomizing 81 participants compared high-dosage versus low-dosage chloroquine diphosphate showed evidence of a beneficial effect of low-dosage chloroquine on all-cause mortality (*p* = 0.024) [51]. One single trial randomizing 110 participants compared colchicine added to standard care versus standard care alone showed evidence of a beneficial effect of standard care on adverse events not considered serious (*p* = 0.003) [50]. One single 3 group trial randomizing 667 participants to hydroxychloroquine with or without azithromycin versus standard care showed evidence of a harmful effect of hydroxychloroquine with azithromycin on adverse events not considered serious (*p* = 0.015) [55].

None of the remaining single trial results showed evidence of a difference on our predefined review outcomes. Two trials did not report the results in a usable way; one trial reported results of the experimental group with a proportion of participants being nonrandomized [57], and the second trial reported the results as per-protocol, and there was participant crossover [43]. Three trials did not report on our review outcomes [45,63,64]. We have contacted all corresponding authors, but we have not been able to obtain outcomes for our analyses from the trialists yet. All trials were assessed as at high risk of bias (**S3 Table**). Characteristics of the trials and their results on the review outcomes can be found in **S2 Table**. Certainty of the evidence was assessed as “low” or “very low” for all outcomes (**S9–S26 Tables**).

### Possible future contributions of ongoing trials

On August 7, 2020, a search on the Cochrane COVID-19 Study Register revealed 1,828 registered randomized clinical trials [13]. From these, 106 different interventions for treatment of COVID-19 patients were identified [13]. The 10 most investigated experimental interventions were hydroxychloroquine (162 trials), convalescent plasma (55 trials), azithromycin (52 trials), lopinavir and ritonavir (40 trials), tocilizumab (33 trials), chloroquine (30 trials), favipiravir (24 trials), remdesivir (15 trials), sarilumab (15 trials), and dexamethasone (13 trials). Eligible trials will continuously be included in the present living systematic review once results become available.

We also identified one press release from the RECOVERY trial that investigates a number of potential treatments for COVID-19 versus standard care, including dexamethasone and hydroxychloroquine [48,53,59,69,70]. The trial also investigates lopinavir–ritonavir (*n* = 1,596) versus standard care (*n* = 3,376). According to the press release, there was no significant difference in the primary outcome of 28-day mortality (22.1% lopinavir–ritonavir versus 21.3% usual care; RR 1.04; 95% CI 0.91–1.18; *p* = 0.58) [70]. There was also no evidence of beneficial effects on the risk of progression to mechanical ventilation or length of hospital stay [70].

## Discussion

We performed the first edition of our living systematic review assessing the beneficial and harmful effects of all treatment interventions for COVID-19. We included 33 trials, randomizing a total of 13,312 participants to an experimental versus a control intervention. Our study showed that dexamethasone and remdesivir might be beneficial for COVID-19 patients, but the certainty of the evidence was low to very low, so more trials are needed. We could reject that hydroxychloroquine is beneficial for COVID-19 in reducing death and serious adverse events at the 20% relative risk reduction level.

We identified one trial randomizing 6,425 participants to dexamethasone versus standard care. This trial showed evidence of a beneficial effect of dexamethasone on all-cause mortality and mechanical ventilation [48]. However, the trial was assessed as at high risk of bias and had methodological limitations (**S2 Table, S3 Table**). First, the trial is described as an adaptive trial, but the reporting of the trial does not comply with the Consolidated Standards of Reporting Trials (CONSORT) extension on adaptive designs [71]. For example, according to the CONSORT extension, trialists should report: “Elements of decision-making rules describing whether, how, and when the proposed trial adaptations will be used during the trial. It involves pre-specifying a set of actions guiding how decisions about implementing the trial adaptations are made given interim observed data (decision rules). It also involves pre-specifying limits or parameters to trigger trial adaptations (decision boundaries)” [71]. However, in the protocol and the statistical analysis plan of the RECOVERY trial, it is stated that the data monitoring committee will request data at a frequency relevant to the emerging data, and no other information is provided. Third, apart from mortality, the trial did not assess other serious or nonserious adverse events as defined by the ICH-GCP guidelines, which limits the validity of the trial. In the trial protocol, it is stated that suspected serious adverse reactions as well as suspected unexpected serious adverse reactions will be recorded, whereas other serious and nonserious adverse events will not be recorded. It is of utmost importance to always assess beneficial and harmful intervention effects on patient-important outcomes. Fourth, the authors emphasize large beneficial effects of dexamethasone on mortality in certain specific subgroups of participants (participants receiving invasive mechanical ventilation or oxygen at baseline). However, these findings should be interpreted with caution because the trialists did not adjust the threshold for significance according to the multiple comparisons including these subgroup analyses, which results in an increased risk of type I errors [72]. Furthermore, these subgroups were not prespecified in any of the trial registries or the trial protocol but appear only in the statistical analysis plan first dated June 9, 2020, a day after the last participant was randomized to the dexamethasone group. Fifth, only 88% in the dexamethasone group and 89% in the standard care group had a confirmed COVID-19 diagnosis at randomization. A total of 12% in the dexamethasone group and 10% in the standard care group had a negative SARS-CoV-2 test result [53]. The relatively large proportion of participants without a confirmed COVID-19 diagnosis included in this trial should be considered when interpreting the trial results. Sixth, the trial was open-label, and hence, the participants, treatment providers, and outcome assessors were not blinded, which might bias the trial results [73]. These limitations need to be considered when interpreting the trial results.

It was possible for us to perform 10 meta-analyses. Meta-analysis showed evidence of a beneficial effect of remdesivir versus placebo on serious adverse events mainly driven by a difference in respiratory failure in the largest trial [32]. Results of the largest trial indicated that remdesivir resulted in a median recovery time of 11 days, compared with a median of 15 days in the placebo group [32]. One single trial compared 5 versus 10 days of remdesivir and showed evidence of a beneficial effect of 5 days of remdesivir on serious adverse events [38]. However, this trial was open-label, did not use blinded outcome assessors, and was assessed at high risk of bias. The effects of remdesivir on other patient-important outcomes are unclear. Furthermore, certainty of the evidence was assessed as “very low” for all-cause mortality and serious adverse events and “low” for nonserious adverse events. Hence, the effects of remdesivir need to be confirmed in future trials at low risk of bias.

Meta-analysis showed no evidence of a difference between hydroxychloroquine versus standard care on all-cause mortality and serious adverse events, and trial sequential analysis showed that we had enough information to reject that hydroxychloroquine reduces the risk of all-cause mortality and serious adverse events with a relative risk reduction of 20%. Meta-analysis showed a harmful effect of hydroxychloroquine on nonserious adverse events. Meta-analysis showed no evidence of a difference between lopinavir–ritonavir versus standard care on serious adverse events and on nonserious adverse events. Meta-analysis showed no evidence of a difference between convalescent plasma versus standard care on all-cause mortality. Because of lack of relevant data, it was not possible to perform other meta-analyses, network meta-analysis, or individual patient data meta-analyses.

A single trial compared the immunomodulator interferon β-1a added to standard care versus standard care alone and showed evidence of a beneficial effect of interferon β-1a on all-cause mortality (*p* = 0.029) [37]. This trial also showed evidence of a beneficial effect of standard care alone on nonserious adverse events (*p* = 0.006). One single trial compared high-dosage with low-dosage chloroquine diphosphate [51] showed evidence of a beneficial effect of low-dosage chloroquine diphosphate on all-cause mortality (*p* = 0.024). One single trial randomizing 110 participants compared colchicine added to standard care versus standard care alone showed evidence of a beneficial effect of standard care on nonserious adverse events (*p* = 0.003). One single 3 group trial randomizing 667 participants to hydroxychloroquine with or without azithromycin versus standard care showed evidence of a harmful effect of hydroxychloroquine with azithromycin on adverse events not considered serious (*p* = 0.015) [55]. However, these single trials were underpowered to confirm or reject realistic intervention effects, and they were assessed as at high risk of bias. Therefore, the trial results should be interpreted with great caution [74].

Our living systematic review has a number of strengths. The predefined methodology was based on The Cochrane Handbook for Systematic Reviews of Interventions [12], the 8-step assessment suggested by Jakobsen and colleagues [18], and trial sequential analysis [23]. Hence, this review considers both risks of systematic errors and risks of random errors. Another strength is the living systematic review design, which allows us to continuously surveil and update the evidence base of existing interventions for treatment of COVID-19, resulting in a decreased timespan from evidence to clinical practice. This is particularly important in this international healthcare crisis, in which a large number of new randomized clinical trials are continuously registered and published.

Our living systematic review also has limitations. The primary limitation is the paucity of trials currently available, and the results from the current meta-analyses are of low or very low certainty. This must be considered when interpreting our meta-analysis results. Secondly, the trials that we succeeded in including were all at risks of systematic errors and random errors. Third, it was not possible to perform the planned individual patient data meta-analyses, network-meta-analysis, or the planned subgroup analyses because of the lack of relevant data. We contacted all trial authors requesting individual patient data, but until now, we only received one dataset [39]. Fourth, we included “time to clinical improvement” as an outcome post hoc. Results of this outcome should be interpreted with caution, because it was not predefined and was chosen after the trials were included in the systematic review. We did not include the outcome “time to clinical improvement” in our protocol because this outcome is poorly defined and if outcome assessors are not adequately blinded, assessments of “improvement” may be biased. Furthermore, time to clinical improvement is not one of the most patient-important outcomes, e.g., most patients would rather survive without complications than recover a few days sooner. We chose to meta-analyze this outcome even though the 2 trials defined this outcome differently, i.e., time to clinical improvement [44] and time to clinical recovery [32] (see “Results”). Hence, this outcome result should be interpreted with caution and should only be considered hypothesis generating. Fifth, all trials differed in the included participants’ disease severity at trial intake (mild, moderate, severe, critically ill), which might result in clinical heterogeneity. We will explore disease severity as a subgroup analysis if this is warranted in later stages of this living systematic review. Sixth, the included trials assessed the outcomes at different time points, which might contribute to increased heterogeneity. Seventh, some data are included from preprints, and these might be subject to change following peer review. Therefore, some results, bias risk assessments, and GRADE summaries might change in later editions of this living systematic review following inclusion of the published peer-reviewed manuscripts.

WHO has recently stopped a clinical trial of the antimalaria drug hydroxychloroquine for treating COVID-19 patients [75]. However, this decision applies only to the conduct of the WHO Solidarity trial and does not apply to the clinical use or research evaluation of hydroxychloroquine in pre- or postexposure prophylaxis in patients exposed to COVID-19. The decision was based on the results of a nonrandomized study published by *The Lancet* on hydroxychloroquine and chloroquine and its effects on hospitalized COVID-19 patients [76]. This study was recently retracted because of several authors questioning the validity of the data used in the study. Based on our data, we can reject that hydroxychloroquine offers benefit to COVID-19 patients.

We have identified 4 important reviews that are comparable to our present project [77–80]. The first is a network meta-analysis just published in *BMJ* [77]. However, at the time of publication, this project did not include all relevant trials [50,52,54–56,59–61,64], including the pivotal trials assessing the effects of hydroxychloroquine [54,55,59].

The second project is a literature review published in *JAMA* using PubMed to identify relevant English-language articles published through March 25, 2020, on pharmacological interventions for COVID-19. The search resulted in 1,315 total articles. This is because the authors also included case reports, case series, and review articles if they lacked randomised clinical trials. Moreover, this review was only a narrative review without meta-analytic methods and trial sequential analysis [78].

The third project is a living mapping of ongoing randomized clinical trials with network meta-analysis on all interventions for COVID-19. The authors are producing and disseminating preliminary results through an open platform [79]. This review includes both prevention and treatment and does not use trial sequential analysis or similar methods to handle problems with multiplicity (repeating updating of meta-analysis, multiple comparisons due to inclusion of multiple interventions, assessing multiple outcomes) [9].

The fourth project is a preprint of a rapid review assessing the effectiveness and safety of antiviral antibody treatments for COVID-19 published in medrXiv [80]. Fifty-four studies were included in the review: 3 controlled trials, 10 cohort studies, 7 retrospective medical record/database studies, and 34 case reports or series. These studies included patients with severe acute respiratory syndrome (SARS, *n* = 33), Middle East respiratory syndrome (MERS, *n* = 16), COVID-19 (*n* = 3), and unspecified coronavirus (n = 2). The most common treatment was ribavirin (*n* = 41), followed by oseltamivir (*n* = 10), and the combination of lopinavir/ritonavir (*n* = 7). The authors conclude that current evidence for the effectiveness and safety of antiviral therapies for COVID-19 is inconclusive and suffers from a lack of well-designed prospective trials. Moreover, this review was only a narrative review without meta-analytic methods and trial sequential analysis [9].

## Conclusions

Our study showed that dexamethasone and remdesivir might be beneficial for COVID-19 patients, but the certainty of the evidence was low to very low, so more trials are needed. We could reject that hydroxychloroquine versus standard care reduces the risk of death and serious adverse events with 20%. Otherwise, no evidence-based treatment for COVID-19 currently exists. This review will continuously inform best practice in treatment and clinical research of COVID-19. There is an urgent need for additional evidence, especially trials assessing the effects of dexamethasone and remdesivir.

### Differences between the protocol and the review

We erroneously reported the adjusted trial sequential analysis alpha as 2% in our published protocol [9]. This has now been corrected to 3.3% according to 2 primary outcomes [18]. Further, we included “time to clinical improvement” as an outcome post hoc.

## Supporting information

S1 Text PRISMA 2009 checklist. PRISMA, Preferred Reporting Items for Systematic Reviews and Meta-Analysis.
(DOC)

S2 Text Search strategies. (DOC)

S1 Table Excluded trials. (DOCX)

S2 Table Characteristics of included studies. (XLSX)

S3 Table Risk of bias assessments. (TIFF)

S4 Table Summary of findings table of dexamethasone versus standard care. (DOCX)

S5 Table Summary of findings table of remdesivir versus placebo. (DOCX)

S6 Table Summary of findings table of hydroxychloroquine versus standard care. (DOCX)

S7 Table Summary of findings table of lopinavir–ritonavir versus standard care. (DOCX)

S8 Table Summary of findings table of convalescent plasma versus standard care. (DOCX)

S9 Table Summary of findings table of favipravir versus umifenovir (arbidol). (DOCX)

S10 Table Summary of findings table of umifenovir (arbidol) versus lopinavir–ritonavir. (DOCX)

S11 Table Summary of findings table of umifenovir (arbidol) versus standard care. (DOCX)

S12 Table Summary of findings table of high-flow versus standard bag-valve mask oxygenation. (DOCX)

S13 Table Summary of findings table of novaferon versus novaferon and lopinavir–ritonavir. (DOCX)

S14 Table Summary of findings table of novaferon and lopinavir–ritonavir versus lopinavir–ritonavir. (DOCX)

S15 Table Summary of Findings table of novaferon versus lopinavir–ritonavir. (DOCX)

S16 Table Summary of findings table of alpha lipotic acid versus placebo. (DOCX)

S17 Table Summary of findings table of baloxavir marboxil versus favipravir. (DOCX)

S18 Table Summary of findings table of baloxavir marboxil versus standard care. (DOCX)

S19 Table Summary of findings table of favipravir versus standard care. (DOCX)

S20 Table Summary of findings table of triple combination versus lopinavir–ritonavir. (DOCX)

S21 Table Summary of findings table of 5 versus 10 days remdesivir. (DOCX)

S22 Table Summary of findings table of interferon beta-1a versus standard care. (DOCX)

S23 Table Summary of findings table of hydroxychloroquine versus chloroquine. (DOCX)

S24 Table Summary of findings table of chloroquine versus standard care. (DOCX)

S25 Table Summary of findings table of colchicine versus standard care. (DOCX)

S26 Table Summary of findings table of high versus low-dosage chloroquine diphosphate. (DOCX)

S27 Table Summary of findings table of methylprednisolone versus standard care. (DOCX)

S28 Table Summary of findings table of hydroxychloroquine plus azithromycin versus standard care. (DOCX)

S29 Table Summary of findings table of hydroxychloroquine versus hydroxychloroquine plus azithromycin. (DOCX)

S30 Table Summary of findings table of intravenous immunoglobin versus standard care. (DOCX)

S31 Table Summary of findings table of hydroxychloroquine versus placebo. (DOCX)

S32 Table Summary of findings table of darunavir/cobicistat/interferon alpha-2b versus interferon alpha-2b. (DOCX)

S33 Table Summary of findings table of ribavirin/interferon alpha versus lopinavir/ritonavir/ interferon alpha. (DOCX)

S34 Table Summary of findings table of ribavirin/interferon alpha versus ribavirin/ lopinavir/ritonavir/ interferon alpha. (DOCX)

S35 Table Summary of findings table of lopinavir/ritonavir/interferon alpha versus ribavirin/ lopinavir/ritonavir/ interferon alpha. (DOCX)

S36 Table Summary of findings table of lincocin versus azitro. (DOCX)

S37 Table Summary of findings table of 99mTc-methyl diphosphonate injection versus standard care. (DOCX)

S38 Table Summary of findings table of interferon alpha-2b plus gamma versus interferon alpha-2b. (DOCX)

S1 Fig Fixed-effect forest plot of remdesivir versus placebo on all-cause mortality. (TIFF)

S2 Fig Forest plot of remdesivir versus placebo on nonserious adverse events. (TIFF)

S3 Fig Trial sequential analysis of remdesivir versus placebo on nonserious adverse events. Trial sequential analysis on remdesivir versus placebo on nonserious adverse events in 2 high risk of bias trials. The DARIS was calculated based on an event rate in the control group of 37%; risk ratio reduction of 20% in the experimental group; type I error of 3.3%; and type II error of 10% (90% power). Diversity was 4%. The required information size was 1,970 participants. The cumulative Z‐curve (blue line) did not cross the trial sequential monitoring boundaries for benefit or harm (red inward sloping lines). The cumulative Z‐curve crossed the inner‐wedge futility line (red outward sloping lines). The green dotted line shows conventional boundaries (alpha 5%). DARIS, diversity‐adjusted required information size; Pc, proportion of participants in control group; RRR, relative risk reduction.
(TIFF)

S4 Fig Forest plot of remdesivir versus placebo on time to clinical improvement/recovery. (DOCX)

S5 Fig Forest plot of hydroxychloroquine versus standard care on all-cause mortality. (TIFF)

S6 Fig Trial sequential analysis of hydroxychloroquine versus standard care on all-cause mortality. Trial sequential analysis on hydroxychloroquine versus standard care on serious adverse events in 7 high risk of bias trials. The DARIS was calculated based on an event rate in the control group of 20.7%; risk ratio reduction of 20% in the experimental group; type I error of 3.3%; and type II error of 10% (90% power). Diversity was 0%. The required information size was 4,123 participants. The cumulative Z‐curve (blue line) did not cross the trial sequential monitoring boundaries for benefit or harm (inward sloping red lines) nor the conventional naive boundaries. The cumulative Z‐curve crossed the inner‐wedge futility line (red outward sloping red lines and the DARIS). The green dotted line shows conventional boundaries (alpha 5%). DARIS, diversity‐adjusted required information size; Pc, proportion of participants in control group; RRR, relative risk reduction.
(TIFF)

S7 Fig Forest plot of hydroxychloroquine versus standard care on serious adverse events. (TIFF)

S8 Fig Trial sequential analysis of hydroxychloroquine versus standard care on serious adverse events. Trial sequential analysis on hydroxychloroquine versus standard care on serious adverse events in 7 high risk of bias trials. The DARIS was calculated based on an event rate in the control group of 21.0%; risk ratio reduction of 20% in the experimental group; type I error of 3.3%; and type II error of 10% (90% power). Diversity was 0%. The required information size was 4,123 participants. The cumulative Z‐curve (blue line) did not cross the trial sequential monitoring boundaries for benefit or harm (inward sloping red lines) nor the conventional naive boundaries. The cumulative Z‐curve crossed the inner‐wedge futility line (red outward sloping red lines and the DARIS). The green dotted line shows conventional boundaries (alpha 5%). DARIS, diversity‐adjusted required information size; Pc, proportion of participants in control group; RRR, relative risk reduction.
(TIFF)

S9 Fig Meta-analysis of hydroxychloroquine versus standard care on nonserious adverse events. (TIFF)

S10 Fig Forest plot of lopinavir/ritonavir versus standard care on serious adverse events. (TIFF)

S11 Fig Trial sequential analysis of lopinavir/ritonavir versus standard care on serious adverse events. Trial sequential analysis on lopinavir–ritonavir versus standard care on serious adverse events in 2 high risk of bias trials. The DARIS was calculated based on an event rate in the control group of 27.6%; risk ratio reduction of 20% in the experimental group; type I error of 3.3%; and type II error of 10% (90% power). Diversity was 0%. The required information size was 2,856 participants. The cumulative Z‐curve (blue line) did not cross the trial sequential monitoring boundaries for benefit or harm (inward sloping red lines) nor the conventional naive boundaries. The cumulative Z‐curve did not cross the inner‐wedge futility line (red outward sloping red lines nor the DARIS). The green dotted line shows conventional boundaries (alpha 5%). DARIS, diversity‐adjusted required information size; Pc, proportion of participants in control group; RRR, relative risk reduction.
(TIFF)

S12 Fig Forest plot of lopinavir/ritonavir versus standard care on nonserious adverse events. (TIFF)

S13 Fig Meta-analysis of convalescent plasma versus standard care on all-cause mortality. (TIFF)

## Abbreviations

CI: confidence interval
COVID-19: coronavirus diease 2019
GRADE: Grading of Recommendation, Assessment, Development, and Evaluation
ICH-GCP: International Conference on Harmonisation–Good Clinical Practice
MERS: Middle East respiratory syndrome
RECOVERY: Randomised Evaluation of COVid-19 thERapY
RR: risk ratio
SARS: severe acute respiratory syndrome
SARS-CoV-2: severe acute respiratory syndrome coronavirus 2
