# Correction: Interventions for treatment of COVID-19: A living systematic review with meta-analyses and trial sequential analyses (The LIVING Project)

**DOI:** 10.1371/journal.pmed.1003517

**Published:** 2020-12-29

**Authors:** The PLOS Medicine Staff

The Fig 1 image in the article PDF is incorrect. Please see the correct Fig 1 here. The publisher apologizes for the error.

**Fig 1:**
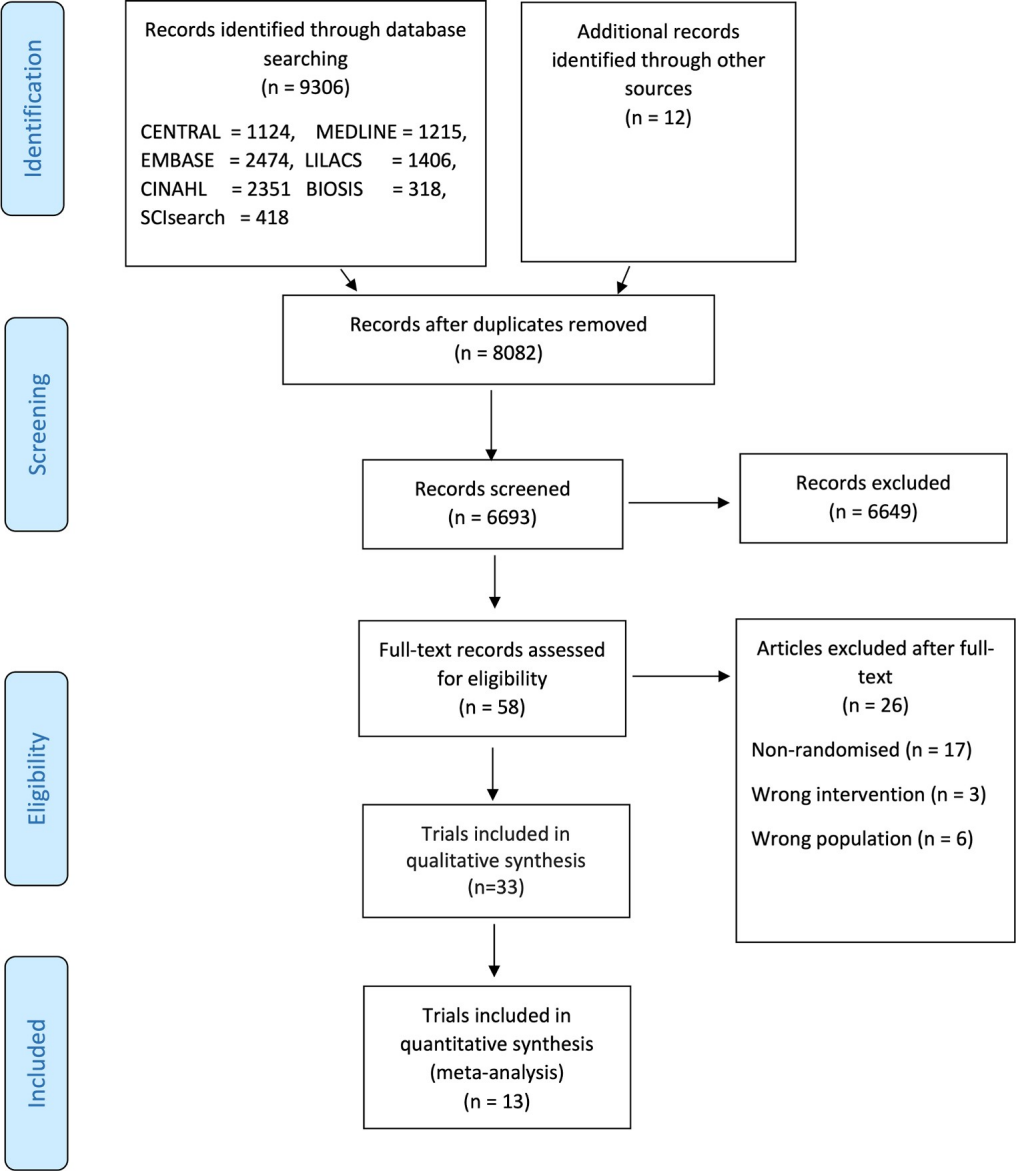
PRISMA flow diagram. BIOSIS, Biosciences Information Services; CENTRAL, Cochrane Central Register of Controlled Trials; CINAHL, Current Index to Nursing and Allied Health Literature; EMBASE, Excerpta Medica database; LILCAS, Latin American and Caribbean Health Sciences Literature; MEDLINE, Medical Literature Analysis and Retrieval System Online; PRISMA, Preferred Reporting Items for Systematic Reviews and Meta-Analysis; SCIsearch, Science Citation Index Search.
