# Supplementary material for: Interventions for treatment of COVID-19: A living systematic review with meta-analyses and trial sequential analyses (The LIVING Project)
Source: PLoS Med. 2020 Sep 17;17(9):e1003293. doi: 10.1371/journal.pmed.1003293 (PMC7498193; doi:10.1371/journal.pmed.1003293)
Supplement: S1 Table — (DOCX) [file pmed.1003293.s003.docx]

# Excluded trials:

| Trial ID | Reason for exclusion |
| --- | --- |
| Boulware 2020 [1] | Wrong population (postexposure prophylaxis) |
| Carlucci 2020 [2] | Not randomised |
| Casadevall 2020 [3] | Not randomised |
| Cattaneo 2020 [4] | Not randomised |
| Cao Y 2020 [5] | Wrong population (suspected COVID-19) |
| ChiCTR2000029954 2020 [6] | Wrong intervention (Chinese medicine) |
| Christensen 2020 [7] | Wrong intervention, and wrong population (teaching) |
| Clariot 2020 [8] | Wrong population (Simulation study, not on patients) |
| Davoodi 2020 [9] | Wrong population (suspected COVID-19) |
| Demidowich 2020 [10] | Wrong population (Non COVID-19) |
| Duan 2020 [11] | Not randomised |
| El-Lababidi 2020 [12] | Not randomised |
| Gautret 2020 [13] | Not randomised |
| Gong 2020 [14] | Not randomised |
| Goyal 2020 [15] | Not randomised |
| Grein 2020 [16] | Not randomised |
| Hu 2020 [17] | Wrong intervention (Chinese medicine) |
| Huang 2020 [18] | Not randomised |
| Liu K 2020 [19] | Not randomised |
| Liu ST 2020 [20] | Not randomised |
| Magagnoli 2020 [21] | Not randomised |
| Mahevas 2020 [22] | Not randomised |
| Pielacinski 2020 [23] | Wrong population (Non COVID-19) |
| Salazar 2020 [24] | Not randomised |
| Somers 2020 [25] | Not randomised |
| Xu 2020 [26] | Not randomised |

1. Boulware DR, Pullen MF, Bangdiwala AS, Pastick KA, Lofgren SM, Okafor EC, et al. A Randomized Trial of Hydroxychloroquine as Postexposure Prophylaxis for Covid-19. New england journal of medicine 2020 jun 03. 2020. doi: 10.1056/NEJMoa2016638. PubMed PMID: 13842397.

2. Carlucci P, Ahuja T, Petrilli CM, Rajagopalan H, Jones S, Rahimian J. Hydroxychloroquine and azithromycin plus zinc vs hydroxychloroquine and azithromycin alone: outcomes in hospitalized COVID-19 patients. medRxiv. 2020:2020.05.02.20080036. doi: 10.1101/2020.05.02.20080036.

3. Casadevall A, Joyner MJ, Pirofski LA. A Randomized Trial of Convalescent Plasma for COVID-19-Potentially Hopeful Signals. JAMA 2020 jun 03. 2020. doi: 10.1001/jama.2020.10218. PubMed PMID: 13842399.

4. Cattaneo M, Morici N. Is thromboprophylaxis with high-dose enoxaparin really necessary for COVID-19 patients? A new "prudent" randomised clinical trial. Blood transfusion. 2020;18(3):237-8. doi: 10.2450/2020.0109-20. PubMed PMID: 13842490; PubMed Central PMCID: PMC2005967395.

5. Cao Y, Wei J, Zou L, Jiang T, Wang G, Chen L, et al. Ruxolitinib in treatment of severe coronavirus disease 2019 (COVID-19): A multicenter, single-blind, randomized controlled trial. Journal of Allergy and Clinical Immunology. 2020;(Epub ahead of print: DOI: 10.1016/j.jaci.2020.05.019). Epub 2020/05/30. doi: 10.1016/j.jaci.2020.05.019. PubMed PMID: 32470486; PubMed Central PMCID: PMCPMC7250105.

6. ChiCTR2000029954. Efficacy and safety of honeysuckle oral liquid in the treatment of novel coronavirus pneumonia (COVID-19): a multicenter, randomized, controlled, open clinical trial. ICTRP. 2020. PubMed PMID: 13841926.

7. Christensen L, Rasmussen CS, Benfield T, Franc JM. A RANDOMIZED TRIAL OF INSTRUCTOR-LED TRAINING VERSUS VIDEO LESSON IN TRAINING HEALTH CARE PROVIDERS IN PROPER DONNING AND DOFFING OF PERSONAL PROTECTIVE EQUIPMENT. Disaster medicine and public health preparedness. 2020:1-15. doi: 10.1017/dmp.2020.56. PubMed PMID: 13251601; PubMed Central PMCID: PMC631375888.

8. Clariot S, Dumain G, Gauci E, Langeron O, Levesque E. Minimising COVID-19 exposure during tracheal intubation by using a transparent plastic box: a randomised prospective simulation study. Anaesthesia critical care & pain medicine 2020 jun 17. 2020. doi: 10.1016/j.accpm.2020.06.005. PubMed PMID: 13987745.

9. Davoodi L, Abedi SM, Salehifar E, Alizadeh-Navai R, Rouhanizadeh H, Khorasani G, et al. Febuxostat therapy in outpatients with suspected COVID-19: A clinical trial. Int J Clin Pract. 2020:e13600. Epub 2020/07/01. doi: 10.1111/ijcp.13600. PubMed PMID: 32603531.

10. Demidowich AP, Levine JA, Apps R, Cheung FK, Chen J, Fantoni G, et al. Colchicine's effects on metabolic and inflammatory molecules in adults with obesity and metabolic syndrome: results from a pilot randomized controlled trial. International journal of obesity 2020 may 27. 2020. doi: 10.1038/s41366-020-0598-3. PubMed PMID: 13842429.

11. Duan K, Liu B, Li C, Zhang H, Yu T, Qu J, et al. Effectiveness of convalescent plasma therapy in severe COVID-19 patients. Proceedings of the National Academy of Sciences of the United States of America. 2020;117(17):9490-6. PubMed PMID: 13586884; PubMed Central PMCID: PMC2005703425.

12. El-Lababidi RM, Mooty M, Bonilla MF, Salem NM. Treatment of severe pneumonia due to COVID-19 with peginterferon alfa 2a. Idcases. 2020;21(no pagination):e00837. doi: 10.1016/j.idcr.2020.e00837. PubMed PMID: 13842453; PubMed Central PMCID: PMC2005982644.

13. Gautret P, Lagier JC, Parola P, Hoang VT, Meddeb L, Mailhe M, et al. Hydroxychloroquine and azithromycin as a treatment of COVID-19: results of an open-label non-randomized clinical trial. International journal of antimicrobial agents. 2020:105949. doi: 10.1016/j.ijantimicag.2020.105949. PubMed PMID: 13251603; PubMed Central PMCID: PMC631332322.

14. Gong Y, Guan L, Jin Z, Chen S, Xiang G, Gao B. Effects of methylprednisolone use on viral genomic nucleic acid negative conversion and CT imaging lesion absorption in COVID-19 patients under 50 years old. Journal of medical virology. 2020. PubMed PMID: 13842697; PubMed Central PMCID: PMC631853763.

15. Goyal DK, Mansab F, Iqbal A, Bhatti S. Early intervention likely improves mortality in COVID-19 infection. Clinical medicine (London, England). 2020. PubMed PMID: 13842719; PubMed Central PMCID: PMC631801518.

16. Grein J, Ohmagari N, Shin D, Diaz G, Asperges E, Castagna A, et al. Compassionate Use of Remdesivir for Patients with Severe Covid-19. New england journal of medicine 2020 apr 10. 2020. doi: 10.1056/NEJMoa2007016. PubMed PMID: 13380846.

17. Hu K, Guan WJ, Bi Y, Zhang W, Li L, Zhang B, et al. Efficacy and safety of Lianhuaqingwen capsules, a repurposed Chinese herb, in patients with coronavirus disease 2019: a multicenter, prospective, randomized controlled trial. Phytomedicine. 2020;(no pagination). PubMed PMID: 13842694; PubMed Central PMCID: PMC2005976431.

18. Huang M, Tang T, Pang P, Li M, Ma R, Lu J, et al. Treating COVID-19 with Chloroquine. Journal of Molecular Cell Biology. 2020. Epub 2020/04/03. doi: 10.1093/jmcb/mjaa014. PubMed PMID: 32236562.

19. Liu K, Zhang W, Yang Y, Zhang J, Li Y, Chen Y. Respiratory rehabilitation in elderly patients with COVID-19: a randomized controlled study. Complementary therapies in clinical practice. 2020;39:101166. doi: 10.1016/j.ctcp.2020.101166. PubMed PMID: 13586742.

20. Liu STH, Lin H-M, Baine I, Wajnberg A, Gumprecht JP, Rahman F, et al. Convalescent plasma treatment of severe COVID-19: A matched control study. medRxiv. 2020:2020.05.20.20102236. doi: 10.1101/2020.05.20.20102236.

21. Magagnoli J, Narendran S, Pereira F, Cummings T, Hardin JW, Sutton SS, et al. Outcomes of hydroxychloroquine usage in United States veterans hospitalized with Covid-19. Medrxiv : the preprint server for health sciences 2020 apr 21. 2020. doi: 10.1101/2020.04.16.20065920. PubMed PMID: 13880598.

22. Mahevas M, Tran V-T, Roumier M, Chabrol A, Paule R, Guillaud C, et al. No evidence of clinical efficacy of hydroxychloroquine in patients hospitalized for COVID-19 infection with oxygen requirement: results of a study using routinely collected data to emulate a target trial. medRxiv. 2020:2020.04.10.20060699. doi: 10.1101/2020.04.10.20060699.

23. Pielacinski K, Pula B, Wroblewski T, Kurylowicz M, Szczepanik AB. Totally extraperitoneal inguinal hernia repair with or without fixation leads to similar results. Outcome of randomized prospective trial. Wideochirurgia I inne techniki maloinwazyjne. 2020;15(1):1-10. PubMed PMID: 13380953; PubMed Central PMCID: PMC2005368148.

24. Salazar E, Perez KK, Ashraf M, Chen J, Castillo B, Christensen PA, et al. Treatment of COVID-19 Patients with Convalescent Plasma in Houston, Texas. Medrxiv : the preprint server for health sciences 2020 may 13. 2020. doi: 10.1101/2020.05.08.20095471. PubMed PMID: 13880621.

25. Somers EC, Eschenauer GA, Troost JP, Golob JL, Gandhi TN, Wang L, et al. Tocilizumab for treatment of mechanically ventilated patients with COVID-19. medRxiv. 2020:2020.05.29.20117358. doi: 10.1101/2020.05.29.20117358.

26. Xu X, Han M, Li T, Sun W, Wang D, Fu B, et al. Effective treatment of severe COVID-19 patients with tocilizumab. Proc Natl Acad Sci U S A. 2020;117(20):10970-5. Epub 2020/05/01. doi: 10.1073/pnas.2005615117. PubMed PMID: 32350134; PubMed Central PMCID: PMCPMC7245089.
