# Supplementary figures and images for: Interventions for treatment of COVID-19: A living systematic review with meta-analyses and trial sequential analyses (The LIVING Project)
Source: PLoS Med. 2020 Sep 17;17(9):e1003293. doi: 10.1371/journal.pmed.1003293 (PMC7498193; doi:10.1371/journal.pmed.1003293)

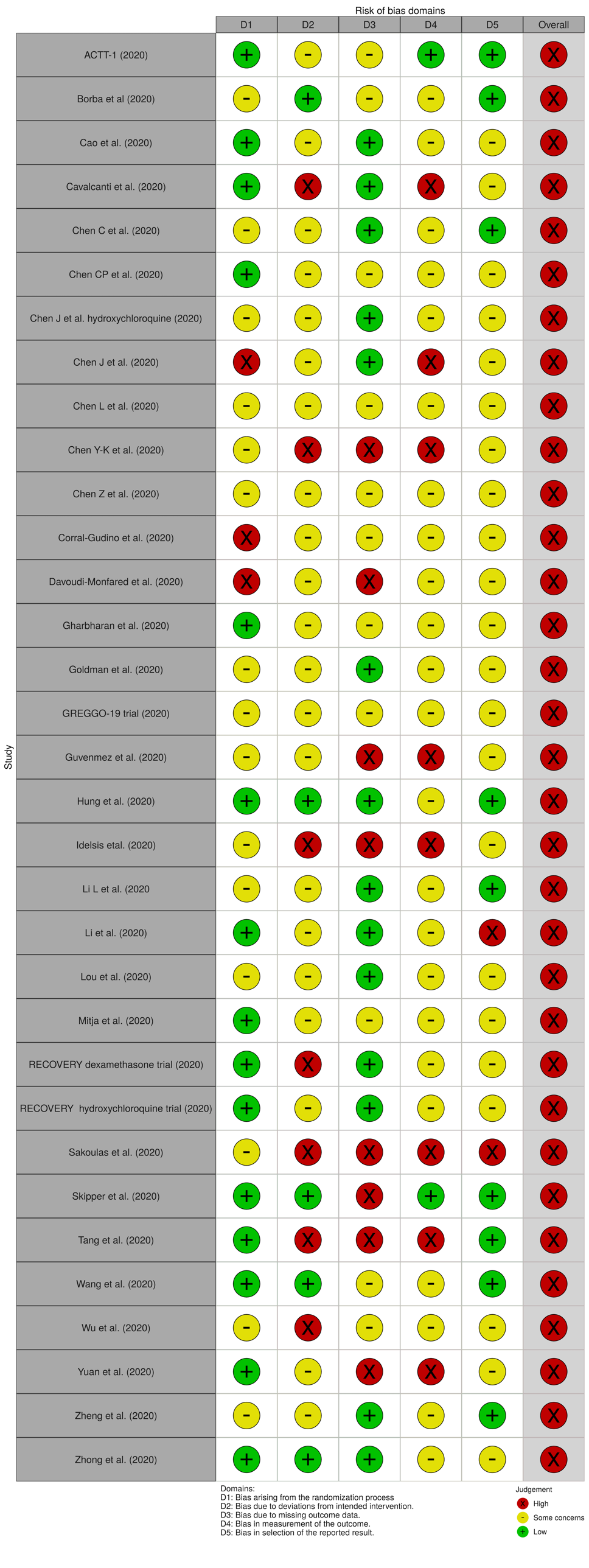

Supplement: S3 Table — (TIFF) [file pmed.1003293.s005.tiff]

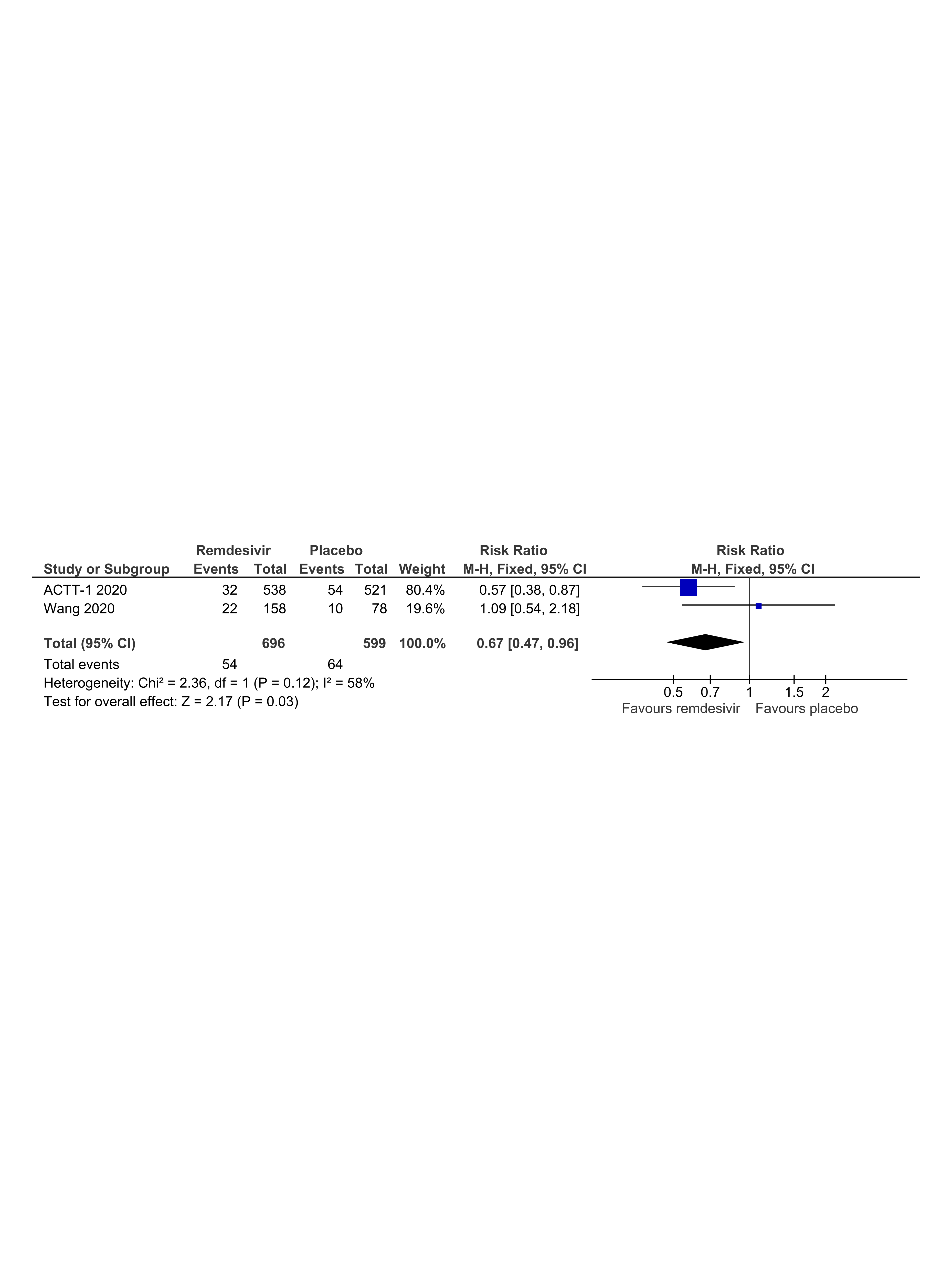

Supplement: S1 Fig — (TIFF) [file pmed.1003293.s041.tiff]

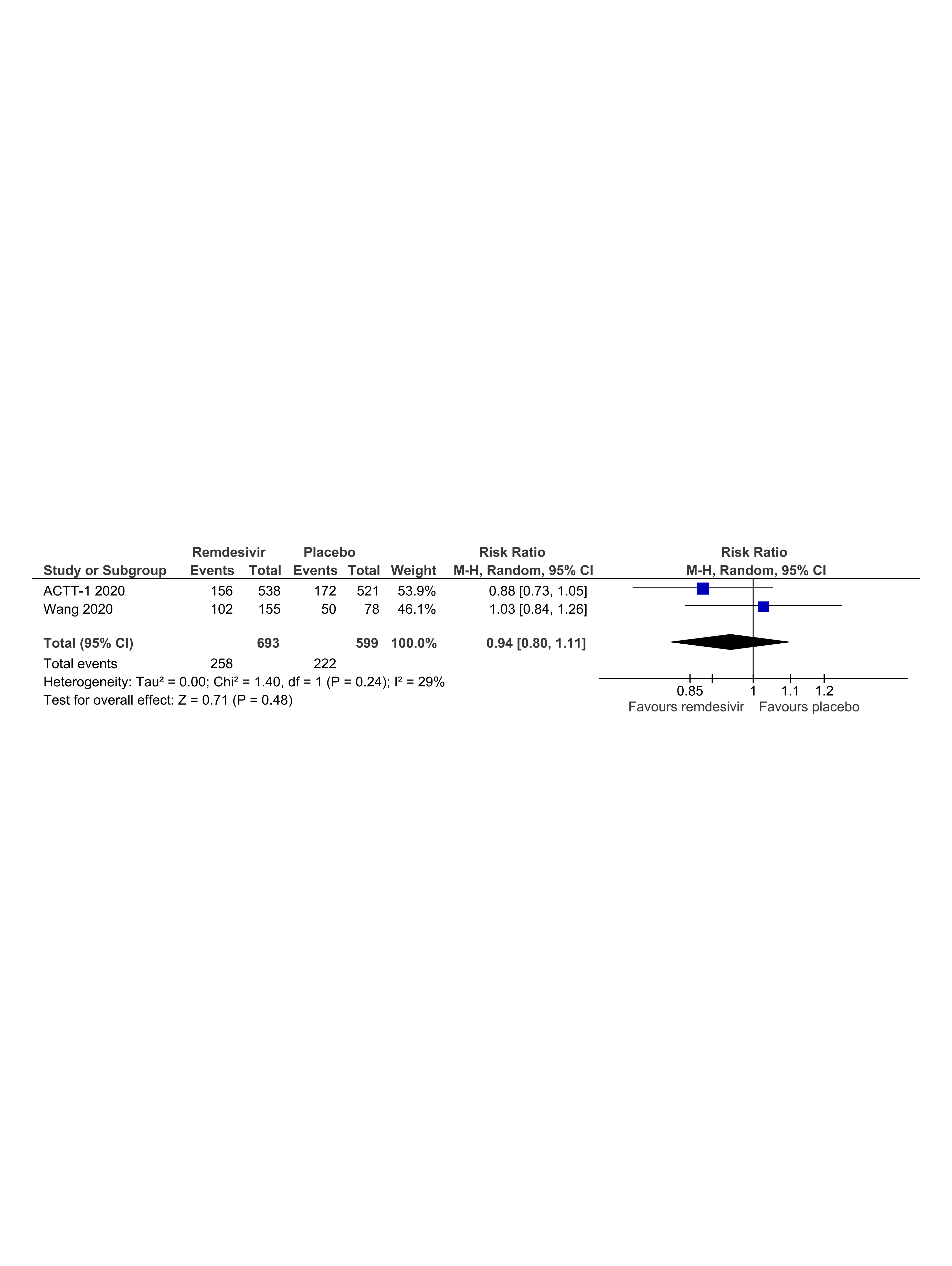

Supplement: S2 Fig — (TIFF) [file pmed.1003293.s042.tiff]

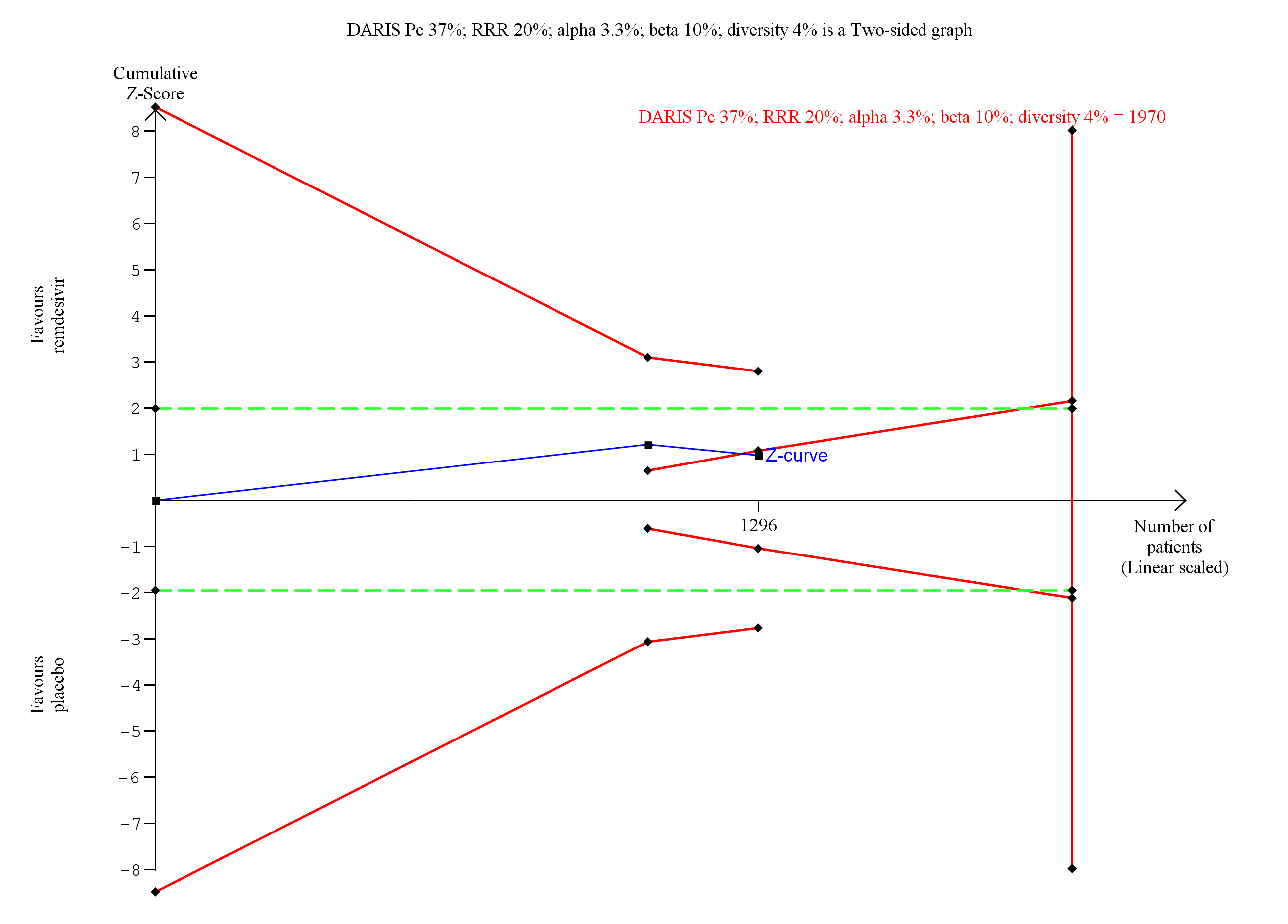

Supplement: S3 Fig — Trial sequential analysis on remdesivir versus placebo on nonserious adverse events in 2 high risk of bias trials. The DARIS was calculated based on an event rate in the control group of 37%; risk ratio reduction of 20% in the experimental group; type I error of 3.3%; and type II error of 10% (90% power). Diversity was 4%. The required information size was 1,970 participants. The cumulative Z‐curve (blue line) did not cross the trial sequential monitoring boundaries for benefit or harm (red inward sloping lines). The cumulative Z‐curve crossed the inner‐wedge futility line (red outward sloping lines). The green dotted line shows conventional boundaries (alpha 5%). DARIS, diversity‐adjusted required information size; Pc, proportion of participants in control group; RRR, relative risk reduction. (TIFF) [file pmed.1003293.s043.tiff]

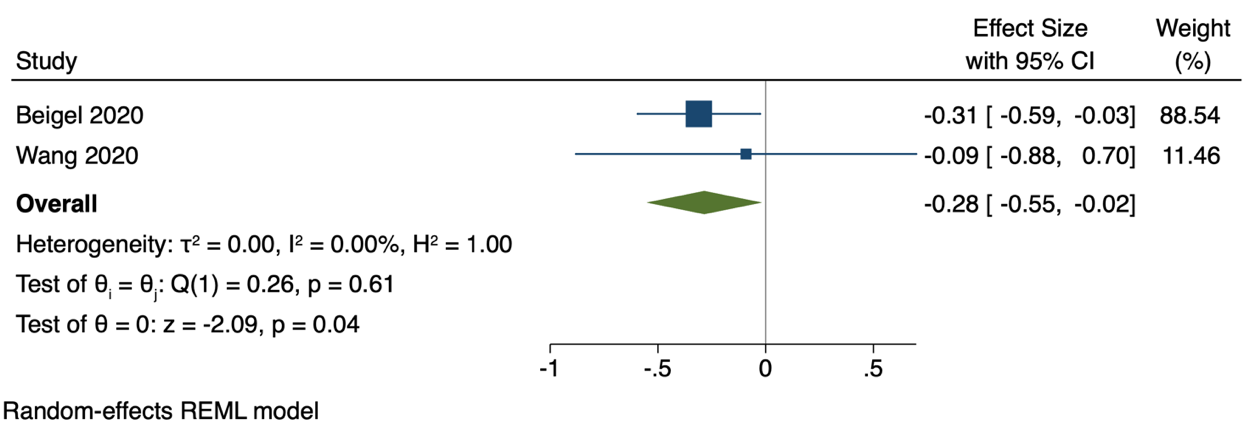

Supplement: S4 Fig — (DOCX) [file pmed.1003293.s044.docx]

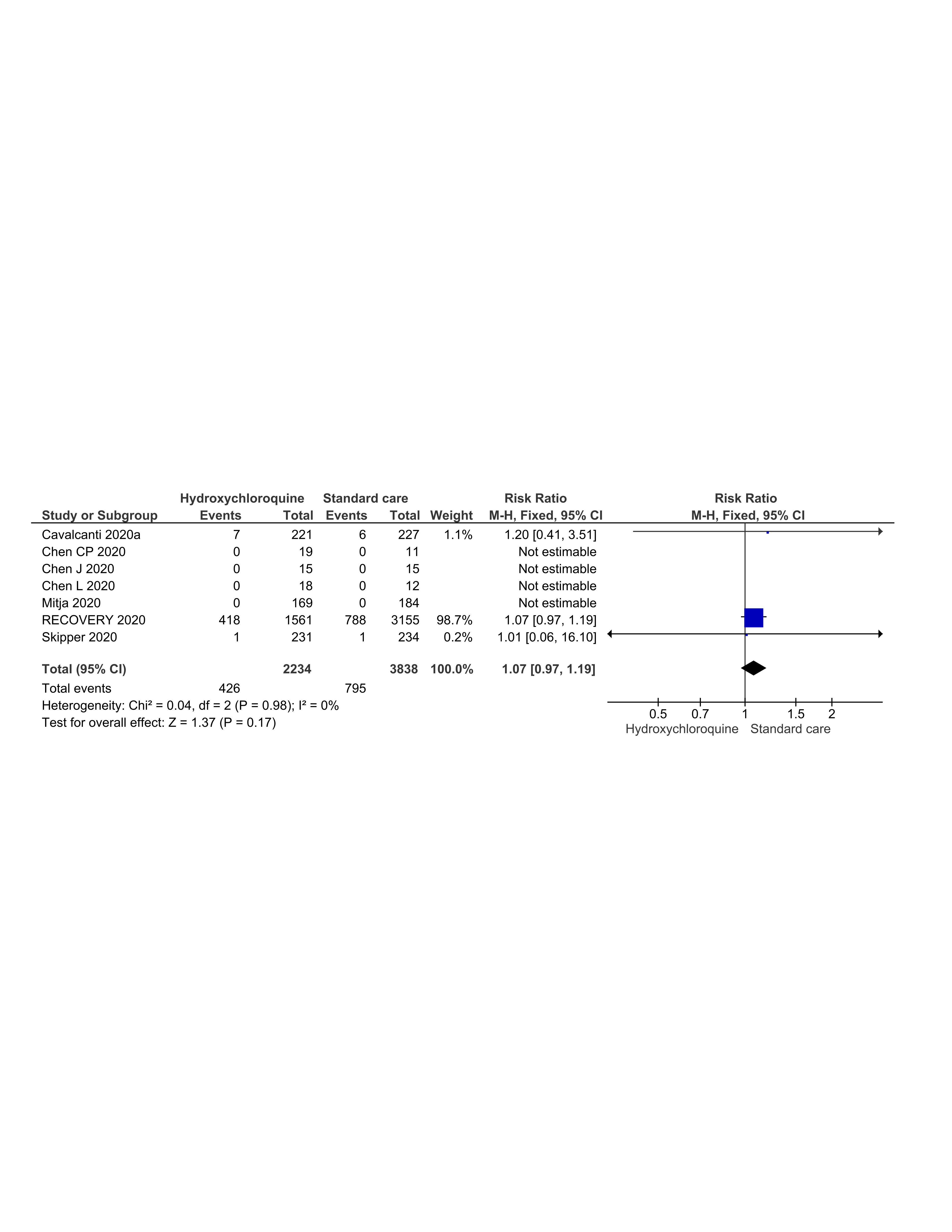

Supplement: S5 Fig — (TIFF) [file pmed.1003293.s045.tiff]

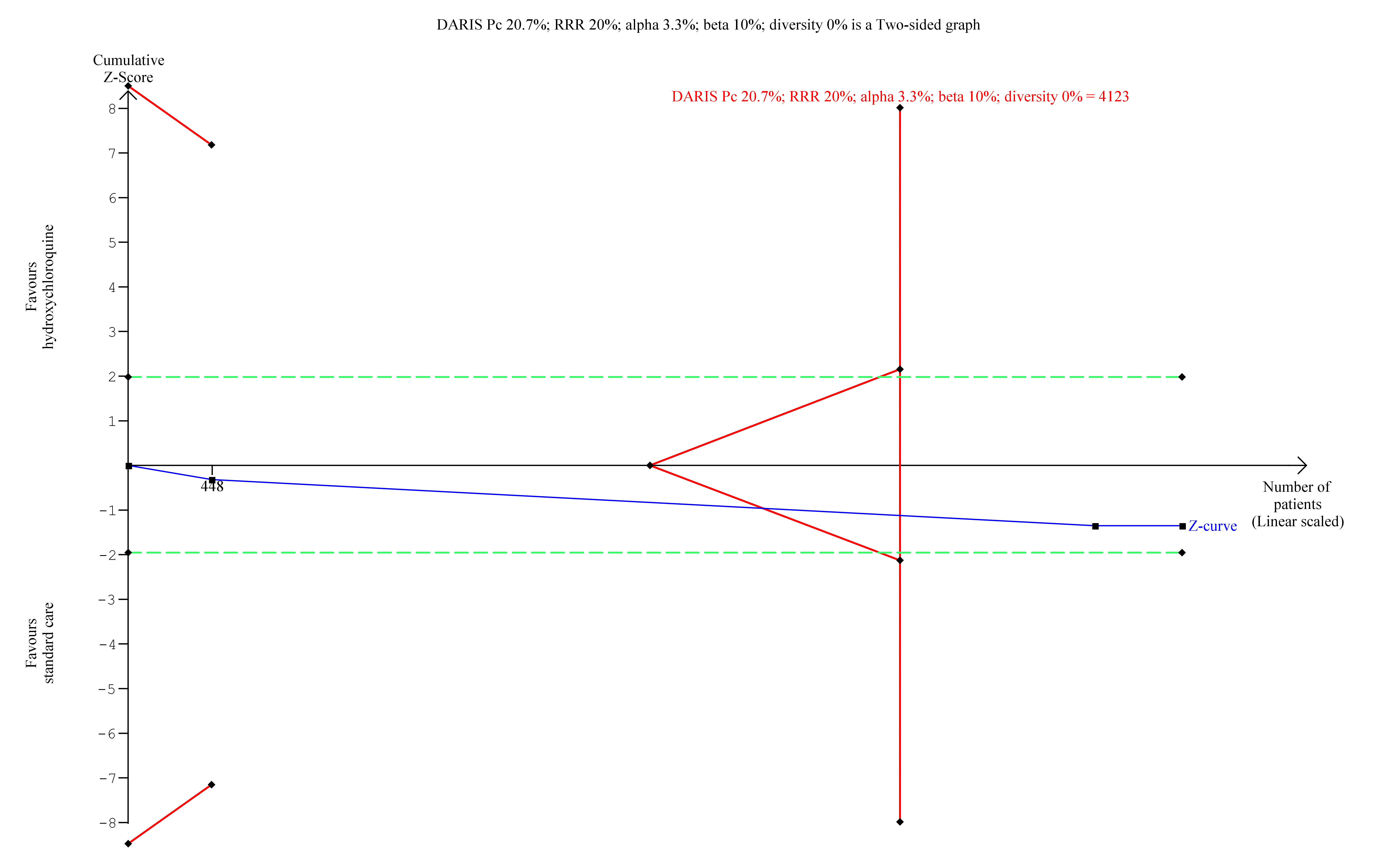

Supplement: S6 Fig — Trial sequential analysis on hydroxychloroquine versus standard care on serious adverse events in 7 high risk of bias trials. The DARIS was calculated based on an event rate in the control group of 20.7%; risk ratio reduction of 20% in the experimental group; type I error of 3.3%; and type II error of 10% (90% power). Diversity was 0%. The required information size was 4,123 participants. The cumulative Z‐curve (blue line) did not cross the trial sequential monitoring boundaries for benefit or harm (inward sloping red lines) nor the conventional naive boundaries. The cumulative Z‐curve crossed the inner‐wedge futility line (red outward sloping red lines and the DARIS). The green dotted line shows conventional boundaries (alpha 5%). DARIS, diversity‐adjusted required information size; Pc, proportion of participants in control group; RRR, relative risk reduction. (TIFF) [file pmed.1003293.s046.tiff]

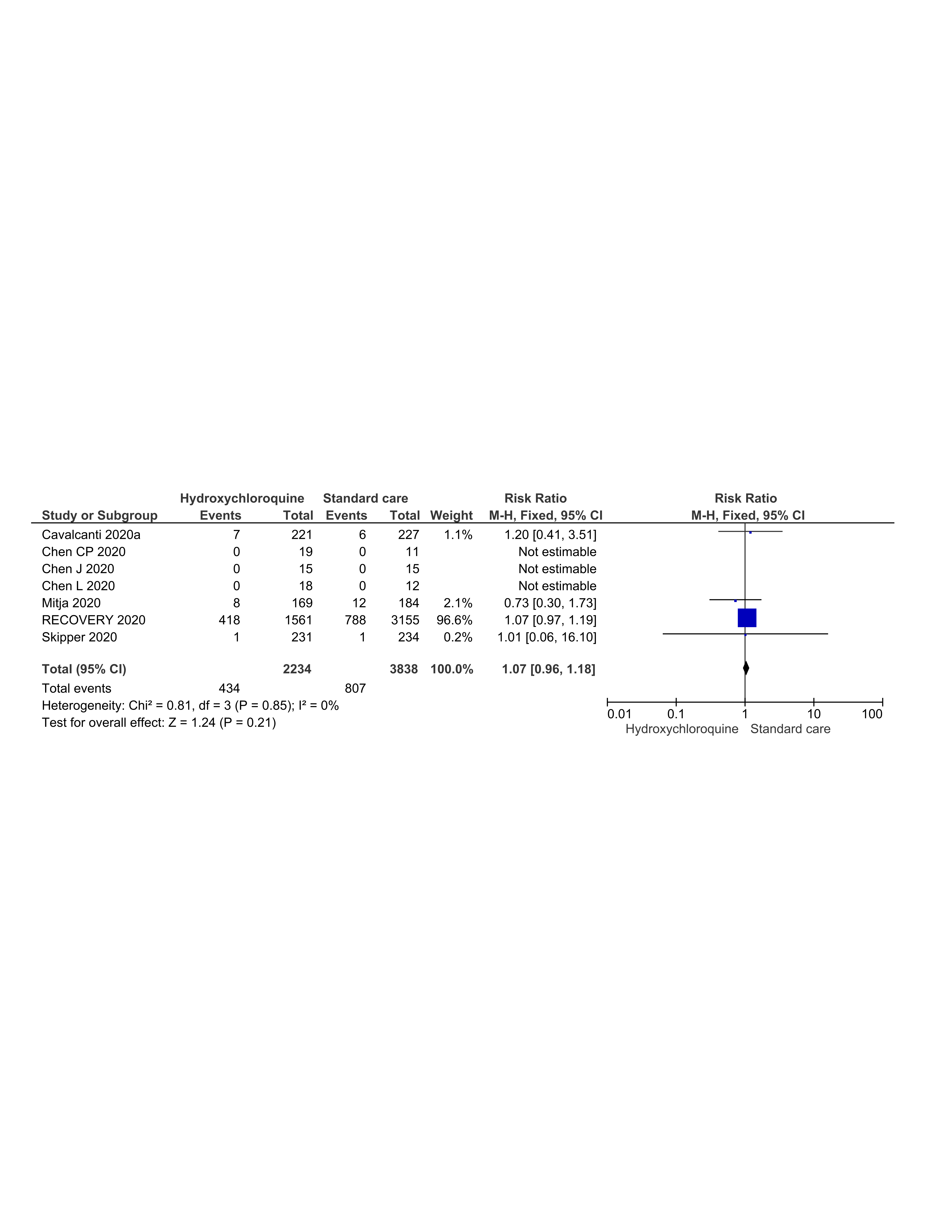

Supplement: S7 Fig — (TIFF) [file pmed.1003293.s047.tiff]

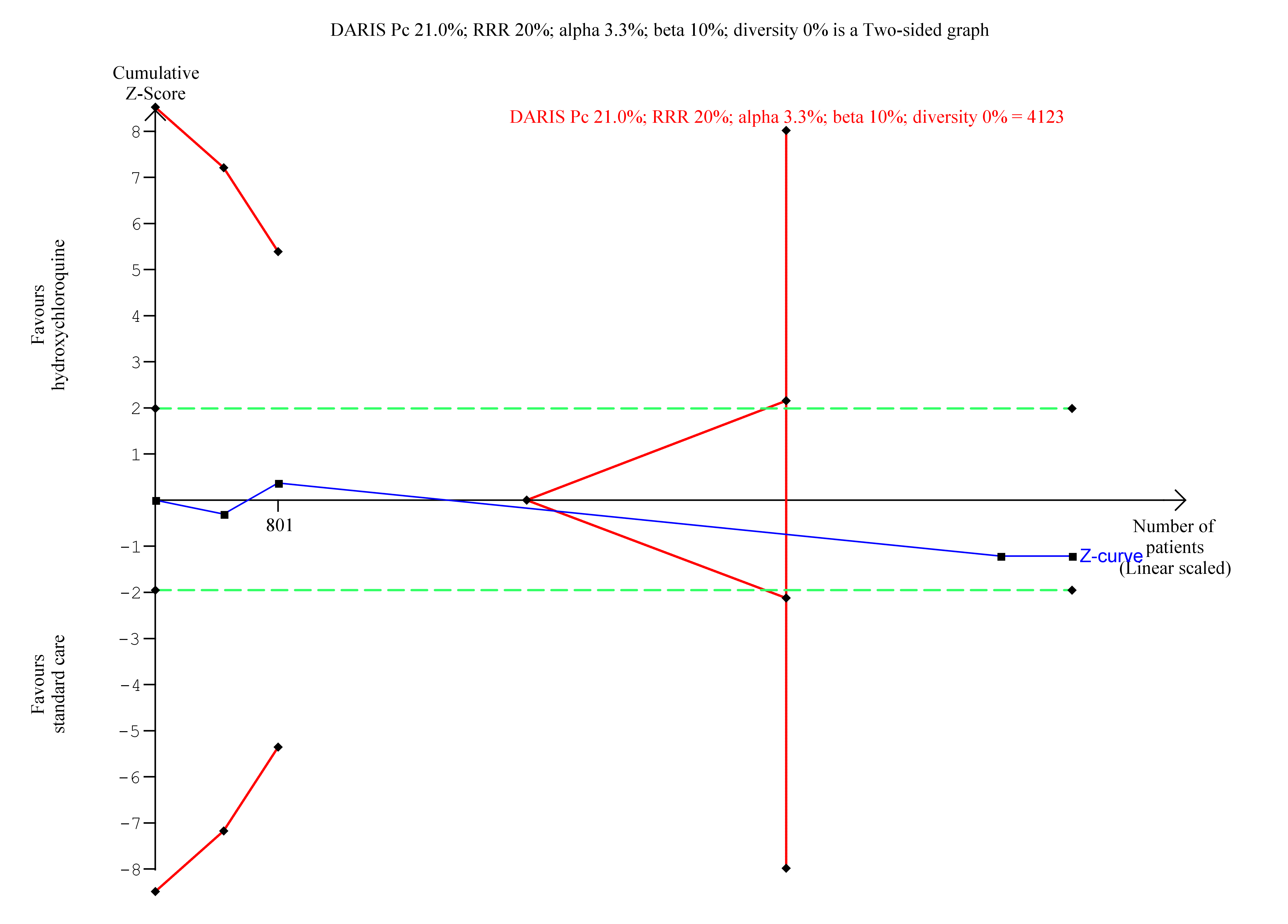

Supplement: S8 Fig — Trial sequential analysis on hydroxychloroquine versus standard care on serious adverse events in 7 high risk of bias trials. The DARIS was calculated based on an event rate in the control group of 21.0%; risk ratio reduction of 20% in the experimental group; type I error of 3.3%; and type II error of 10% (90% power). Diversity was 0%. The required information size was 4,123 participants. The cumulative Z‐curve (blue line) did not cross the trial sequential monitoring boundaries for benefit or harm (inward sloping red lines) nor the conventional naive boundaries. The cumulative Z‐curve crossed the inner‐wedge futility line (red outward sloping red lines and the DARIS). The green dotted line shows conventional boundaries (alpha 5%). DARIS, diversity‐adjusted required information size; Pc, proportion of participants in control group; RRR, relative risk reduction. (TIFF) [file pmed.1003293.s048.tiff]

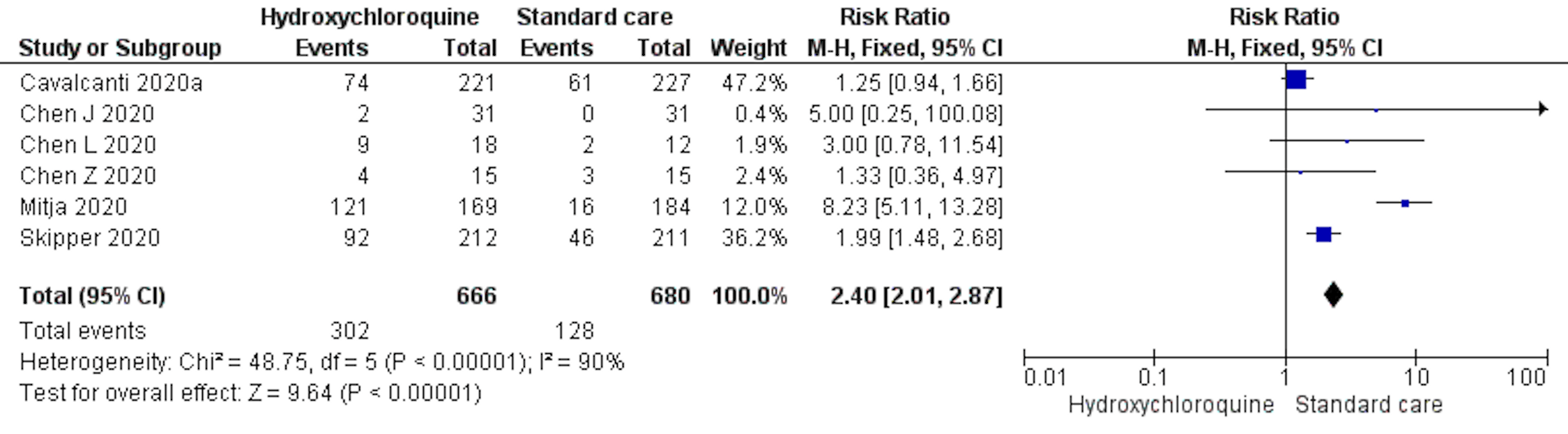

Supplement: S9 Fig — (TIFF) [file pmed.1003293.s049.tiff]

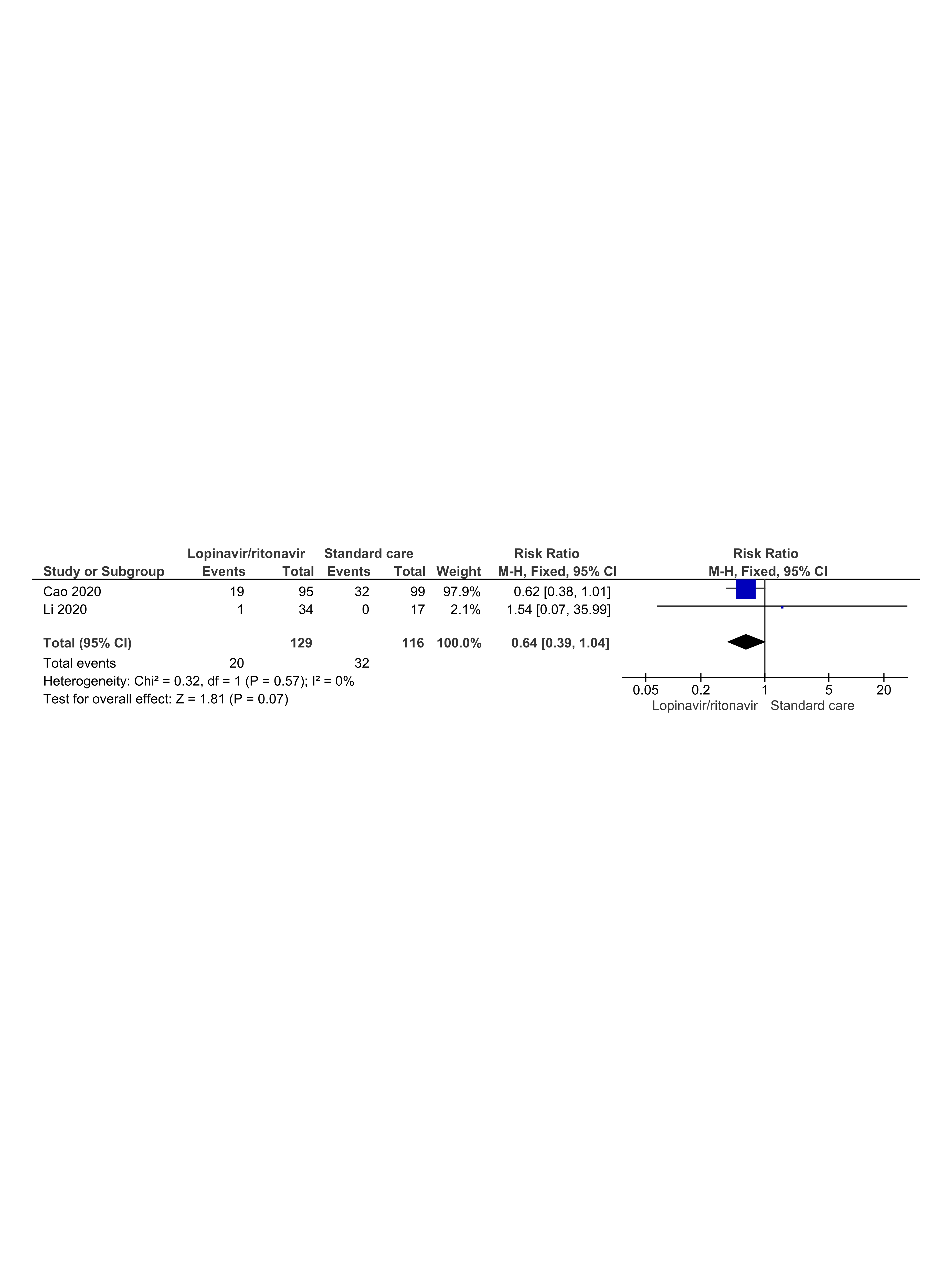

Supplement: S10 Fig — (TIFF) [file pmed.1003293.s050.tiff]

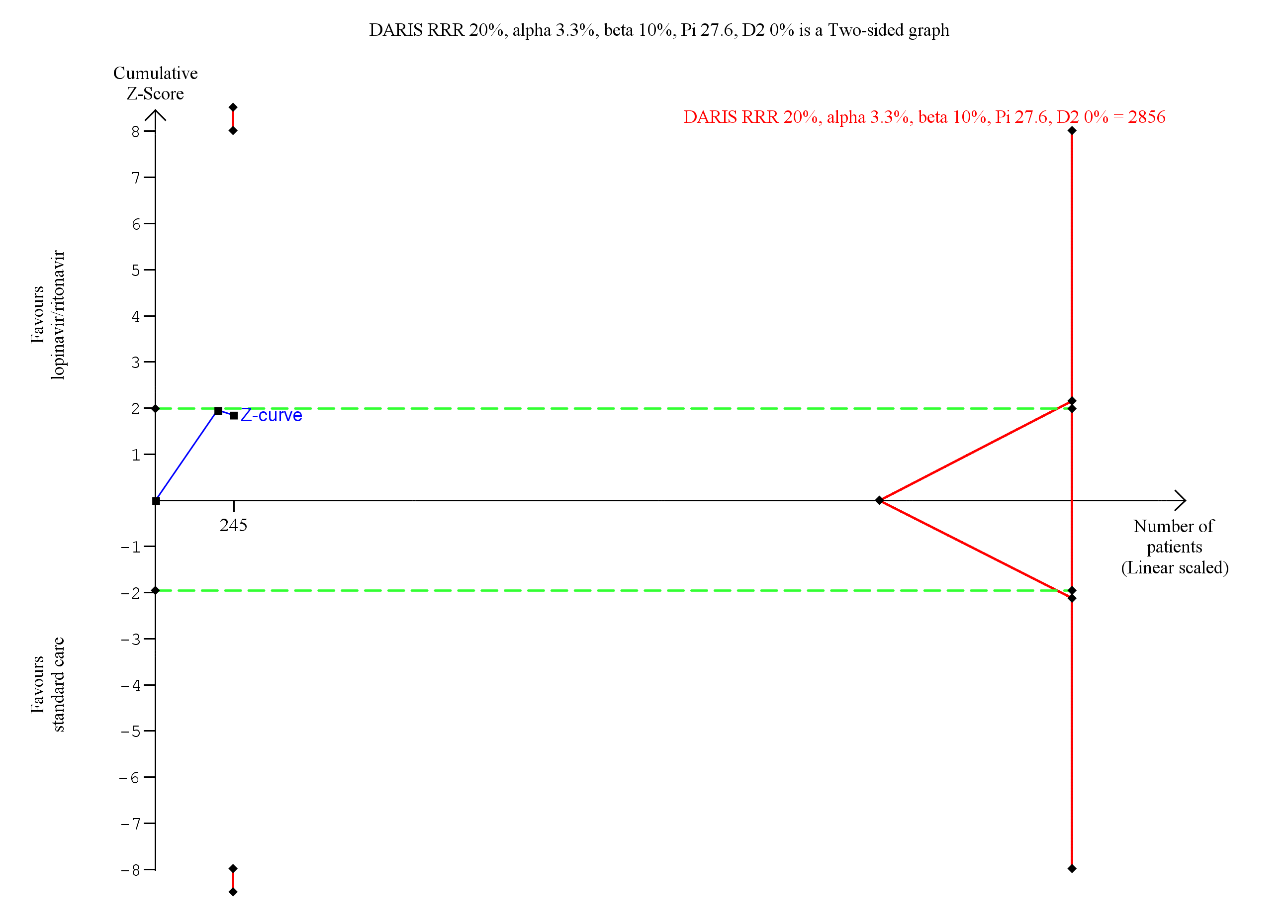

Supplement: S11 Fig — Trial sequential analysis on lopinavir–ritonavir versus standard care on serious adverse events in 2 high risk of bias trials. The DARIS was calculated based on an event rate in the control group of 27.6%; risk ratio reduction of 20% in the experimental group; type I error of 3.3%; and type II error of 10% (90% power). Diversity was 0%. The required information size was 2,856 participants. The cumulative Z‐curve (blue line) did not cross the trial sequential monitoring boundaries for benefit or harm (inward sloping red lines) nor the conventional naive boundaries. The cumulative Z‐curve did not cross the inner‐wedge futility line (red outward sloping red lines nor the DARIS). The green dotted line shows conventional boundaries (alpha 5%). DARIS, diversity‐adjusted required information size; Pc, proportion of participants in control group; RRR, relative risk reduction. (TIFF) [file pmed.1003293.s051.tiff]

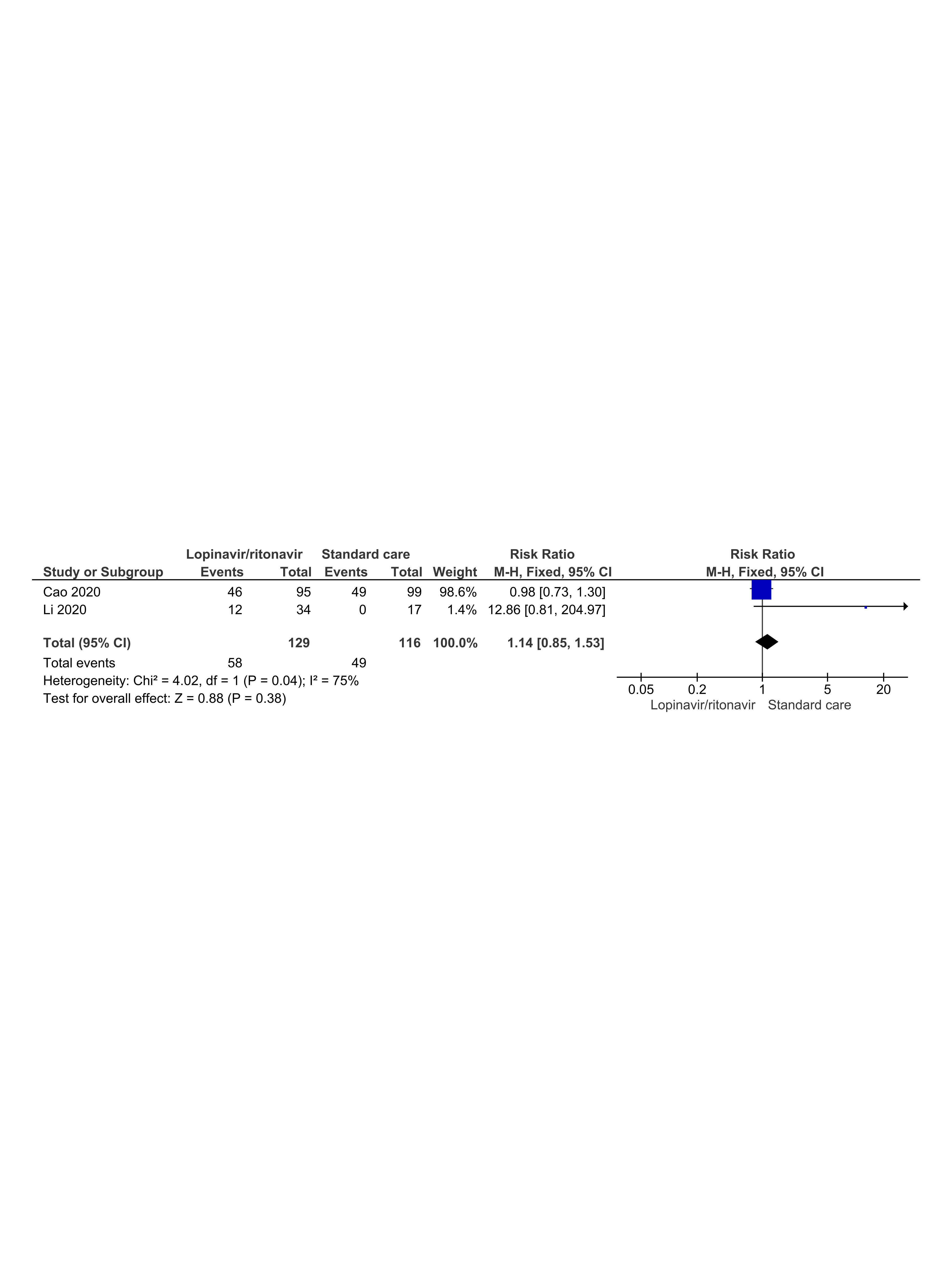

Supplement: S12 Fig — (TIFF) [file pmed.1003293.s052.tiff]

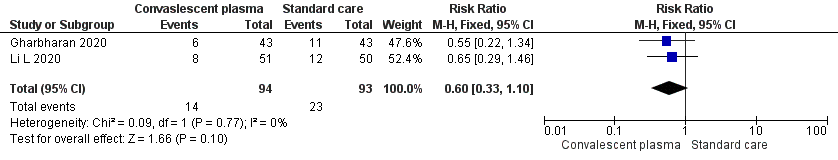

Supplement: S13 Fig — (TIFF) [file pmed.1003293.s053.tiff]
