# Supplementary material for: Interventions for treatment of COVID-19: A living systematic review with meta-analyses and trial sequential analyses (The LIVING Project)
Source: PLoS Med. 2020 Sep 17;17(9):e1003293. doi: 10.1371/journal.pmed.1003293 (PMC7498193; doi:10.1371/journal.pmed.1003293)
Supplement: S6 Table — (DOCX) [file pmed.1003293.s008.docx]

S6 Summary of Findings

| **Hydroxychloroquine compared with standard care for COVID-19** | | | | | | |
| --- | --- | --- | --- | --- | --- | --- |
| **Patients or population:** Anyone with a diagnosis of COVID-19  **Setting:** Any setting  **Intervention:** Hydroxychloroquine  **Comparison:** Standard care | | | | | | |
| **Outcomes** | **Anticipated absolute effects* (95% CI)** | | **Relative effect (95% CI)** | **No of participants (studies)** | **Certainty of the evidence (GRADE)** | **Comments** |
|  | **Risk with standard care** | **Risk with**  **Hydroxychloroquine** |  |  |  |  |
| **All-cause mortality**  *Follow-up: mean 18.9 days* | 207 per 1,000 | **222 per 1,000**  (201 to 246) | **1.07**  (0.97 to 1.19) | 6072  (7 RCTs) | ⨁⨁◯◯ LOW ^a^ |  |
| **Serious adverse events**  *Follow-up: mean 18.9 days* | 210 per 1,000 | **225 per 1,000**  (202 to 248) | **1.07**  (0.96 to 1.18) | 6072  (7 RCTs) | ⨁⨁◯◯ LOW ^a^ |  |
| **Admission to intensive care** | - | - | - | - | - | Outcome not yet measured or reported |
| **Mechanical ventilation** | - | - | - | - | - | Outcome not yet measured or reported |
| **Renal replacement therapy** | - | - | - | - | - | Outcome not yet measured or reported |
| **Quality of Life** | - | - | - | - | - | Outcome not yet measured or reported |
| **Non-serious adverse events**  *Follow-up: mean 14.3 days* | 188 per 1,000 | **451 per 1,000**  (395 to 540) | **RR 2.40** (2.01 to 2.87) | 1346  (6 RCTs) | ⨁◯◯◯ VERY LOW ^a,b^ |  |
| *The risk in the intervention group (and its 95% confidence interval) is based on the assumed risk in the comparison group and the relative effect of the intervention (and its 95% CI).  **RR:** Risk ratio; **CI:** Confidence interval; **GRADE:** GRADE Working Group grades of evidence | | | | | | |
| **GRADE Working Group grades of evidence**  **High certainty:** We are very confident that the true effect lies close to that of the estimate of the effect **Moderate certainty:** We are moderately confident in the effect estimate: The true effect is likely to be close to the estimate of the effect, but there is a possibility that it is substantially different **Low certainty:** Our confidence in the effect estimate is limited: The true effect may be substantially different from the estimate of the effect **Very low certainty:** We have very little confidence in the effect estimate: The true effect is likely to be substantially different from the estimate of effect | | | | | | |

**Explanations**

a. Downgraded 2 for risk of bias

b. Downgraded 2 for heterogeneity (I^2^ = 90%)
